# Effect of a Web-Based Management Guide on Risk Factors in Patients With Type 2 Diabetes and Diabetic Kidney Disease: A JADE Randomized Clinical Trial

**DOI:** 10.1001/jamanetworkopen.2022.3862

**Published:** 2022-03-25

**Authors:** Juliana C. N. Chan, Yotsapon Thewjitcharoen, Thy Khue Nguyen, Alexander Tan, Yook-Chin Chia, Chii-Min Hwu, Du Jian, Thep Himathongkam, Kim-Leng Wong, Yun-Mi Choi, Roberto Mirasol, Mafauzy Mohamed, Alice P. S. Kong, Ronald C. W. Ma, Elaine Y. K. Chow, Risa Ozaki, Vanessa Lau, Amy W. C. Fu, Eun-Gyoung Hong, Kun-Ho Yoon, Chiu-Chi Tsang, Eric S. H. Lau, Lee-Ling Lim, Andrea O. Y. Luk

**Affiliations:** 1 Asia Diabetes Foundation, Shatin, Hong Kong Special Administrative Region (SAR), China; 2 Department of Medicine and Therapeutics, The Chinese University of Hong Kong, Prince of Wales Hospital, Shatin, Hong Kong SAR, China; 3 Hong Kong Institute of Diabetes and Obesity, The Chinese University of Hong Kong, Prince of Wales Hospital, Shatin, Hong Kong SAR, China; 4 Li Ka Shing Institute of Health Sciences, The Chinese University of Hong Kong, Prince of Wales Hospital, Shatin, Hong Kong SAR, China; 5 Diabetes and Thyroid Center, Theptarin Hospital, Bangkok, Thailand; 6 Medic Medical Center, Ho Chi Minh City, Vietnam; 7 Sunway Medical Centre, Selangor, Malaysia; 8 Department of Medical Sciences, Sunway University, Selangor, Malaysia; 9 Department of Primary Care Medicine, Faculty of Medicine, University of Malaya, Kuala Lumpur, Malaysia; 10 Department of Medicine, Taipei Veterans General Hospital, Taipei, Taiwan; 11 Department of Endocrinology, The Fourth Affiliated Hospital of China Medical University, Shenyang, China; 12 Department of Internal Medicine, Dongtan Sacred Heart Hospital, Hallym University College of Medicine, Hwaseong, Korea; 13 Section of Endocrinology, Diabetes and Metabolism, St Luke's Medical Center, Quezon City, Philippines; 14 Department of Medicine, Universiti Sains Malaysia, Kota Bahru, Malaysia; 15 Department of Endocrinology and Metabolism, Seoul St Mary's Hospital, The Catholic University of Korea, Seoul, Korea; 16 Department of Medicine, Alice Ho Miu Ling Nethersole Hospital, Tai Po, Hong Kong SAR, China; 17 Department of Medicine, Faculty of Medicine, University of Malaya, Kuala Lumpur, Malaysia

## Abstract

**Importance:**

Diabetic kidney disease (DKD) and its comorbidities can be prevented by treating multiple targets. Technology-assisted team-based care with regular feedback and patient empowerment can improve the attainment of multiple targets and clinical outcomes in patients with type 2 diabetes, but the effects of this intervention on patients with DKD are unclear.

**Objective:**

To evaluate the effect of the Joint Asia Diabetes Evaluation (JADE) web portal, nurse reminders, and team-based care on multiple risk factors in patients with DKD.

**Design, Setting, and Participants:**

This 12-month multinational, open-label randomized clinical trial was conducted between June 27, 2014, and February 19, 2019, at 13 hospital-based diabetes centers in 8 countries or regions in Asia. All patients who participated had DKD. The intention-to-treat data analysis was performed from April 7 to June 30, 2020.

**Interventions:**

Patients were randomized in a 1:1:1 ratio at each site to usual care, empowered care, or team-based empowered care. All patients underwent a JADE web portal–guided structured assessment at baseline and month 12. Patients in the usual care and empowered care groups received a medical follow-up. Patients in the empowered care group also received a personalized JADE report and nurse telephone calls every 3 months. Patients in the team-based empowered care group received additional face-to-face reviews every 3 months from a physician-nurse team.

**Main Outcomes and Measures:**

The primary outcome was the proportion of patients who attained multiple treatment targets (defined as ≥3 of 5 targets: HbA_1c_ level <7.0% [53 mmol/mol], blood pressure <130/80 mm Hg, low-density lipoprotein cholesterol level <1.8 mmol/L, triglyceride level <1.7 mmol/L, and/or persistent use of renin-angiotensin-aldosterone system inhibitors).

**Results:**

A total of 2393 patients (mean [SD] age, 67.7 [9.8] years; 1267 men [52.9%]) were randomized to the usual care group (n = 795), empowered care group (n = 802), and team-based empowered care group (n = 796). At baseline, 34.7% patients (n = 830) were on 3 treatment targets. On intention-to-treat analysis, the team-based empowered care group had the highest proportion of patients who had further increase in attainment of multiple treatment targets (within-group differences: usual care group, 3.9% [95% CI, 0.0%-7.8%]; empowered care group, 1.3% [95% CI, −2.8% to 5.4%]; team-based empowered care group, 9.1% [95% CI, 4.7%-13.5%]). The team-based empowered care group was more likely to attain multiple treatment targets than the usual care group (risk ratio [RR], 1.17; 95% CI, 1.00-1.37) and the empowered care group (RR, 1.25; 95% CI, 1.06-1.48) after adjustment for site. Compared with the group that did not attain multiple treatment targets, the group that attained multiple treatment targets reported a lower incidence of cardiovascular, kidney, and cancer events (8.4% [n = 51] vs 14.5% [n = 134]; *P* = .004). Analysis of the per-protocol population yielded similar results.

**Conclusions and Relevance:**

This trial found that technology-assisted team-based care for 12 months improved the attainment of multiple treatment targets as well as empowerment in patients with DKD.

**Trial Registration:**

ClinicalTrials.gov Identifier: NCT02176278

## Key Points

**Question:**

What is the effect of using the Joint Asia Diabetes Evaluation (JADE) web portal, nurse reminders, and team-based care on reducing multiple risk factors in patients with diabetic kidney disease (DKD)?

**Findings:**

In this randomized clinical trial involving 2393 patients with DKD, those randomized to the empowered care group received a personalized report and nurse telephone calls every 3 months in addition to usual care, whereas the team-based empowered care group received additional face-to-face reviews every 3 months from a physician-nurse team. Compared with the usual care and empowered care groups, the team-based empowered care group was more likely to attain at least 3 of the 5 treatment targets.

**Meaning:**

The findings from this trial suggest that data-driven, team-based care improves patient empowerment and decreases multiple risk factors in patients with DKD.

## Teaser

This randomized clinical trial examines the use of a web portal combined with a physician-and-nurse care team approach in improving patient involvement and treatment outcomes for type 2 diabetes and diabetic kidney disease.

## Introduction

Diabetes is the leading cause of chronic kidney disease and end-stage kidney disease worldwide.^1^ Many patients with diabetic kidney disease (DKD) die of a cardiovascular event before the initiation of kidney replacement therapy.^2^ In clinical trials, control of blood pressure (BP),^3^ blood glucose,^4^ and blood cholesterol level^5^ as well as use of renin-angiotensin-aldosterone system (RAAS) inhibitors,^6^ sodium-glucose cotransporter 2 inhibitors,^7,8^ and finerenone^9^ have been shown to improve cardiovascular and kidney outcomes and survival in patients with DKD.

In clinical practice, target attainment rates are low, often because of delayed intervention and suboptimal self-management.^10,11,12^ For example, in Asia, 30% to 40% of patients with DKD attained recommended glycemic and BP targets, and 50% were prescribed RAAS inhibitors and statins.^13,14^ A meta-analysis of 181 randomized clinical trials (RCTs) involving 135 112 patients with type 2 diabetes found that team-based care, patient education, self-management support, and improved patient-clinician communication had the largest effect sizes in reducing cardiometabolic risk factors, especially in low-resource areas such as Asia,^15^ although there is a lack of evidence in DKD.

The importance of early detection, risk stratification, and timely management of DKD calls for more comprehensive implementation strategies.^16^ In this multicenter RCT that was conducted in Asia, we aimed to evaluate the effects of the Joint Asia Diabetes Evaluation (JADE) web portal, nurse reminders, and team-based care on multiple risk factors in patients with DKD. We hypothesized that the JADE web portal–assisted team-based care along with regular feedback and patient empowerment is a viable strategy for improving treatment target attainment and outcomes in this patient population (see trial protocol in Supplement 1).

## Methods

This multinational, open-label, 3-group RCT was conducted from June 27, 2014, to February 19, 2019, at 13 hospital-based diabetes centers in 8 countries or regions in Asia (The Fourth Affiliated Hospital of China Medical University, China; Alice Ho Miu Ling Nethersole Hospital, Hong Kong; Prince of Wales Hospital, Hong Kong; Universiti Sains Malaysia, Malaysia; University of Malaya Medical Centre, Malaysia; St Luke's Medical Center, Philippines; Seoul St Mary’s Hospital, South Korea; Hallym University Dongtan Sacred Heart Hospital, South Korea; Taipei Veterans General Hospital, Taiwan; Theptarin Hospital, Thailand; and Medic Medical Center, Vietnam) (eTable 1 in Supplement 2). Each site was either given a grant, which was equivalent to an 18-month nurse salary at that center, to recruit 300 patients or was paid pro rata. The RCT complied with the Declaration of Helsinki^17^ and received approval from the local institutional review boards of the participating centers. All participating patients signed a written informed consent form before study enrollment. The present study followed the Consolidated Standards of Reporting Trials (CONSORT) reporting guideline.

### Participants, Randomization, and Masking

Between June 27, 2014, and March 21, 2018, we screened 2421 patients. At each site, patients self-reported their own race and ethnicity (Chinese, Filipino, Indian, Korean, Malay, Thai, and Vietnamese) and were verified by attending clinicians.

All eligible patients had type 2 diabetes, which was defined as nonketotic presentation or no insulin requirement within 1 year of diagnosis. Given that an estimated glomerular filtration rate (eGFR)^18^ was not automatically reported in some sites, we defined DKD as either an eGFR of less than 60 mL/min/1.73m^2^ or serum creatinine with a 30% or more upper reference limit to facilitate recruitment. Because of the small number of patients with a low eGFR in a primary care setting, in January 2015, we included patients with an eGFR of 65 to 90 mL/min/1.73m^2^ and macroalbuminuria (urinary albumin to creatinine ratio ≥25 mg/mmol). Exclusion criteria included an eGFR of less than 15 mL/min/1.73m^2^, the need for kidney replacement therapy, inability to give consent, and life-threatening illnesses or conditions that were considered unsuitable by the investigators at each site.

**Figure 1.**
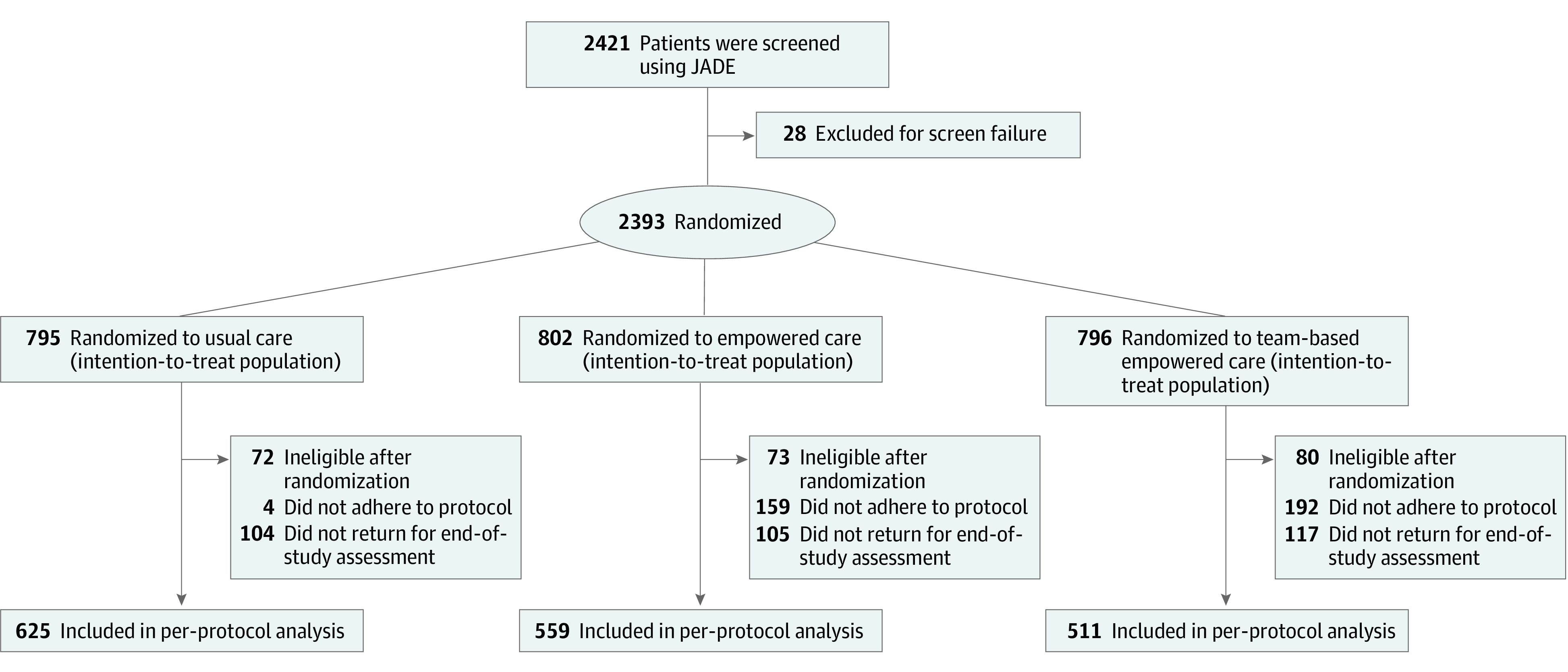
CONSORT Study Flow Diagram The number of patients in the per-protocol population might not add up because some patients were excluded from the per-protocol analysis.

Eligible patients at each study site were randomized in a 1:1:1 ratio to usual care, empowered care, or team-based empowered care (Figure 1). Computer-generated assignment codes were put in sealed, opaque, and consecutively numbered envelopes and then opened by non–study personnel at the site. Patients, investigators, and nurses were not blinded according to the design of the study.

### Study Procedures

The JADE web portal was developed and has been managed by the Asia Diabetes Foundation since 2007, and its functions have been described elsewhere.^11^ This portal consists of templates that guide the comprehensive assessment of eye, feet, blood, and urine and documentation of demographic characteristics, socioeconomic status, lifestyle, medical history, physical assessments, laboratory measurements, and medications using a standardized case report form. The portal incorporates validated risk equations for risk stratification and issues a personalized report with automated decision support for patients and physicians. The aim of this report is to empower self-management and care that is tailored to the needs of the patient. Systematic data collection is the basis of a register, which can be created to inform practice and policies.^11,19^

During a 1.5-day workshop that consisted of lectures, demonstrations, and role-playing, participating physicians and nurses were instructed to treat multiple targets for patients who were randomized to the team-based empowered care group. All sites were given an operating manual with instructions on care organization, care protocols, evaluation procedures, use of the JADE web portal, and interpretation and explanation of the JADE personalized reports. In general, messages in the patient report focus on self-management and treatment adherence, whereas messages in the physician report focus on early intervention, use of organ-protective drugs, and referral for patient education.^19,20^ The Asia Diabetes Foundation conducted online site monitoring using the JADE web portal and issued newsletters every 3 to 6 months that covered recruitment status, completeness of data entry, and reminders on study procedures.

All randomized patients underwent a JADE portal–guided assessment at baseline and the end of the study at month 12. Those in the usual care and empowered care groups received medical follow-up according to practice at the site. Those in the empowered care group also received a personalized report with a nurse explanation during a face-to-face visit and telephone reminders on adherence to clinic visits, medication, and self-management from a nurse every 3 months. In addition to these procedures, those in the team-based empowered care group attended a clinic visit every 3 months that was managed by a team of 1 nurse and 1 physician. Laboratory tests at the sites were used without changes in the assays during the study period.

### Outcome Definition

The primary outcome was the proportion of patients who attained multiple (at least 3 of 5) treatment targets: a hemoglobin A_1c_ (HbA_1c_) level less than 7.0% (to convert to the proportion of total hemoglobin, multiply by 0.01 or 53 mmol/mol), BP less than 130/80 mm Hg, a low-density lipoprotein (LDL) cholesterol level less than 1.8 mmol/L, a triglyceride level less than 1.7 mmol/L, and persistent use of RAAS inhibitors. Because diabetes is associated with multiple morbidities, including cancer,^21^ we defined the secondary outcome as a composite of incident cardiovascular, kidney, and cancer events (eTable 2 in Supplement 2) in patients who did or did not attain multiple treatment targets. Other outcomes included self-monitoring of blood glucose at least once per week, regular exercise at least 3 times per week, and/or adherence to a balanced diet (yes or no) in the past 3 months.

We used a standardized case report form to capture the incidence of cardiovascular, kidney, and cancer events at the reassessment at month 12. The investigators at each site provided a narrative to describe the new events, including hospitalization period, clinical presentation, diagnosis, and outcome. Occurrence of death during the study period was reported. An endocrinologist and a statistician who were not involved in the trial adjudicated all events.

### Statistical Analysis

The sample size was calculated using PASS, version 11 (NCSS). The premise of the RCT was that reducing multiple risk factors would be beneficial for cardiovascular and kidney events.^3,22,23^ In the multicenter Structured vs Usual Care on Renal Endpoints in Type 2 Diabetes (SURE) Study conducted in Hong Kong in early 2000,^24^ the structured care group managed by a physician-and-nurse team was 3 times more likely to attain at least 3 targets and was associated with a 50% risk reduction in cardiovascular and kidney events after 2 years. In Asia, less than 10% of patients with DKD attained multiple targets.^13^ We estimated that 10% of patients in the usual care group, 20% in the empowered care group, and 30% in the team-based empowered care group would attain at least 3 targets after a 12-month intervention. A sample size of 1000 patients in each group had 95% power to test the primary outcome with a 2-sided likelihood-ratio test that was adjusted for multiple comparisons using Bonferonni correction (type I error set at 1%).

All randomized patients were included in the intention-to-treat analysis. Protocol adherence, as documented in the JADE web portal, was defined as follows: 2 or fewer nurse contacts at baseline and month 12 for the usual care group, additional 3 telephone contacts by nurses over 12 months for the empowered care group, and 6 or more clinic visits and/or nurse telephone contacts by the same physician-and-nurse team over 12 months for the team-based empowered care group. Per-protocol analyses included patients who fulfilled all inclusion and exclusion criteria, adhered to prespecified study procedures, and returned for reassessment at month 12.

We used the χ^2^ test; Fisher exact test; unpaired, 2-tailed *t* test; and analysis of variance for between-group comparisons, as appropriate. We also used McNemar test for within-group comparisons. Poisson regression model was used to derive the risk ratios (RRs) and 95% CIs for the attainment of multiple treatment targets in the team-based empowered care group vs the usual care and empowered care groups, which were adjusted for site (model 1) and for site and baseline insulin use (model 2) attributed to between-group differences. Patients with a history of a cardiovascular, kidney, and cancer event were excluded in the regression analysis for the secondary outcome. In the intention-to-treat analysis, missing data were handled by multiple imputation by chained equations with 20 imputations.^25^ The analyses after imputations were combined by the Rubin rule.^26^

The data analysis was performed from April 7 to June 30, 2020, using R, version 4.1.2 (R Foundation for Statistical Computing).^27^ A 2-sided *P* < .05 was considered to be statistically significant.

## Results

### Participant Characteristics

After excluding 28 patients with type 1 diabetes, diabetes of unknown type, or out-of-range eGFR (Figure 1), we randomized a total of 2393 patients to the usual care group (n = 795), empowered care group (n = 802), and team-based empowered care group (n = 796). The site in the Philippines and 1 site in South Korea did not randomize any patients because of an administrative delay (eTable 1 in Supplement 2).

The randomized cohort had a mean (SD) age of 67.7 (9.8) years and was composed of 1267 men (52.9%) and 1126 women (47.1%). At baseline, these patients had a mean (SD) duration of diabetes of 16.4 (9.8) years, and 89.6% of patients (n = 2143) had an eGFR of 15 to 65 mL/min/1.73m^2^, 36.2% (n = 866) had macroalbuminuria, and 30.9% (n = 738) had a history of cardiovascular disease. Lipid-lowering drugs were prescribed in 77.4% of patients (n = 1851), RAAS inhibitors in 69.6% (n = 1665), and insulin in 47.4% (n = 1135), and 34.7% of patients (n = 830) had at least 3 targets at baseline. A total of 40% to 80% of patients (n = 951 to 1928) performed self-monitoring and/or regular exercise and/or dietary adherence. All 3 groups had similar profiles, except for higher insulin use in those in the team-based empowered care group (Table 1).

**Table 1.** Baseline Clinical Characteristics of Patients by Group Randomization

Variable	No. (%)			
	Total (N = 2393)	Usual care group (n = 795)	Empowered care group (n = 802)	Team-based empowered care group (n = 796)
Sociodemographic characteristics				
Age, mean (SD), y	67.7 (9.8)	67.9 (9.9)	67.5 (10.2)	67.5 (9.4)
Men	1267 (52.9)	425 (53.5)	415 (51.7)	427 (53.6)
Women	1126 (47.1)	370 (46.5)	387 (48.3)	369 (46.4)
Race and ethnicity^a^				
Chinese	1068 (44.6)	363 (45.6)	355 (44.3)	350 (43.9)
Indian	190 (8.0)	57 (7.2)	62 (7.8)	71 (8.9)
Korean	355 (14.8)	118 (14.9)	120 (15.0)	117 (14.7)
Malay	295 (12.3)	98 (12.3)	100 (12.5)	97 (12.2)
Thai	161 (6.7)	53 (6.7)	55 (6.9)	53 (6.7)
Vietnamese	316 (13.2)	103 (13.0)	105 (13.1)	108 (13.5)
Other^b^	5 (0.3)	2 (0.2)	4 (0.4)	0
≥College-level education	484 (20.2)	150 (18.8)	166 (20.7)	169 (21.2)
Smoking status				
Current	216 (9.0)	77 (9.7)	68 (8.5)	70 (8.8)
Previous	446 (18.7)	145 (18.2)	156 (19.5)	146 (18.3)
Diabetic and metabolic profile, mean (SD)				
Diabetes duration, y	16.4 (9.8)	16.1 (9.8)	16.5 (9.8)	16.6 (9.9)
Age at diagnosis, y	51.2 (11.6)	51.8 (11.6)	51.0 (11.8)	50.9 (11.4)
BMI	26.9 (4.8)	26.8 (4.5)	27.1 (4.9)	26.9 (4.8)
Waist circumference, cm				
Men	95.8 (11.0)	95.5 (10.6)	96.1 (11.5)	95.9 (10.9)
Women	92.8 (11.8)	93.0 (11.7)	92.7 (11.8)	92.7 (11.9)
BP, mm Hg				
Systolic	139.0 (18.6)	139.0 (19.5)	138.0 (17.6)	138.0 (18.5)
Diastolic	74.3 (11.1)	74.2 (11.1)	74.4 (10.7)	74.2 (11.6)
HbA_1c_ level, %	7.9 (1.6)	7.9 (1.7)	7.8 (1.6)	7.9 (1.7)
HbA_1c_ level, mmol/mol	62.5 (17.8)	62.4 (18.3)	62.1 (17.2)	63.0 (18.1)
Fasting plasma glucose level, mmol/L	8.2 (3.4)	8.3 (3.4)	8.1 (3.3)	8.1 (3.5)
Total cholesterol level, mmol/L	4.4 (1.1)	4.4 (1.1)	4.4 (1.1)	4.3 (1.1)
Triglyceride level, mmol/L	1.9 (1.3)	1.9 (1.3)	1.9 (1.4)	1.9 (1.3)
HDL-cholesterol level, mmol/L	1.2 (0.4)	1.2 (0.4)	1.2 (0.4)	1.2 (0.4)
LDL-cholesterol level, mmol/L	2.4 (1.1)	2.3 (0.9)	2.4 (1.1)	2.4 (1.2)
eGFR, mL/min/1.73m^2^	49.8 (16.3)	50.6 (16.5)	49.0 (15.7)	49.6 (16.8)
Urinary ACR, mg/mmol	62.6 (148.0)	58.6 (131.0)	61.2 (143.0)	68.2 (149.0)
General obesity^c^	1466 (61.3)	495 (62.3)	495 (61.7)	477 (59.9)
Hypertension^d^	2249 (94.0)	744 (93.6)	755 (94.1)	750 (94.3)
Dyslipidemia^e^	2287 (95.6)	762 (95.9)	770 (95.9)	755 (94.9)
Complications at baseline				
eGFR <65 mL/min/1.73m^2^	2143 (89.6)	706 (88.8)	731 (91.1)	707 (88.8)
Macroalbuminuria	866 (36.2)	283 (35.6)	293 (36.6)	290 (36.4)
CAD	506 (21.1)	161 (20.3)	168 (20.9)	177 (22.2)
Stroke	200 (8.4)	69 (8.7)	64 (8.0)	67 (8.4)
PAD	161 (6.7)	54 (6.8)	60 (7.5)	47 (5.9)
Any CVD	738 (30.9)	241 (30.3)	245 (30.6)	252 (31.7)
CHF	95 (4.0)	27 (3.4)	39 (4.9)	29 (3.6)
Cancer	119 (5.0)	35 (4.4)	42 (5.2)	42 (5.3)
Diabetic retinopathy	469 (19.6)	159 (20.0)	145 (18.0)	165 (20.7)
Peripheral neuropathy	598 (25.0)	188 (23.7)	209 (26.1)	201 (25.3)
Medication use at baseline				
RAAS inhibitors	1665 (69.6)	550 (69.2)	551 (68.7)	564 (70.9)
BP-lowering drugs	2003 (83.7)	668 (84.0)	659 (82.2)	676 (84.9)
Lipid-lowering drugs	1851 (77.4)	626 (78.7)	614 (76.6)	611 (76.8)
Noninsulin glucose-lowering drugs	1977 (82.6)	668 (84.0)	650 (81.0)	659 (82.8)
Insulin	1135 (47.4)	347 (43.6)	387 (48.3)	401 (50.4)
Diabetes self-care in past 3 mo				
SMBG at least once per wk	1379 (57.6)	441 (55.5)	476 (59.3)	462 (58.1)
Physical exercise at least 3 times per wk	951 (39.7)	314 (39.5)	326 (40.6)	311 (39.1)
Adherence to balanced diet	1928 (80.6)	637 (80.1)	661 (82.5)	631 (79.2)
At least 2 self-care activities	1487 (64.6)	507 (63.8)	529 (66.0)	510 (64.1)
Metabolic targets				
HbA_1c_ level <7.0% (53 mmol/mol)	783 (32.7)	278 (34.9)	260 (32.4)	245 (30.8)
BP <130/80 mm Hg	671 (28.1)	228 (28.7)	213 (26.5)	230 (28.9)
LDL-cholesterol level <1.8 mmol/L	636 (26.6)	208 (26.2)	203 (25.3)	225 (28.2)
Triglyceride level <1.7 mmol/L	1289 (53.9)	434 (54.5)	430 (53.6)	425 (53.4)
≥3 treatment targets	830 (34.7)	273 (34.3)	276 (34.4)	283 (35.5)

Abbreviations: ACR, albumin to creatinine ratio; BMI, body mass index (calculated as weight in kilograms divided by height in meters squared); BP, blood pressure; CAD, coronary artery disease; CHF, congestive heart failure; CVD, cardiovascular disease; eGFR, estimated glomerular filtration rate; HbA_1c_, glycated hemoglobin; HDL, high-density lipoprotein; LDL, low-density lipoprotein; PAD, peripheral artery disease; RAAS, renin-angiotensin-aldosterone system; SMBG, self-monitoring of blood glucose.

SI conversion factor: to convert HbA_1c_ to the proportion of total hemoglobin, multiply by 0.01.

^a^ Race and ethnicity were self-reported and verified by attending clinicians.

^b^ Other ethnicity categories were not reported by the participating sites.

^c^ General obesity was defined as BMI greater than or equal to 25.

^d^ Hypertension was defined as BP of 130/80 mm Hg or higher and/or use of BP-lowering drugs.

^e^ Dyslipidemia was defined as LDL-cholesterol level greater than or equal to 1.8 mmol/L and/or use of lipid-lowering drugs.

At month 12, 87.4% of patients (n = 691) in the usual care group, 86.9% (n = 697) in the empowered care group, and 85.3% (n = 679) in the team-based empowered care group underwent reassessment (Figure 1). Compared with those who returned for reassessment, those who did not return had worse risk factor control (mean [SD] HbA_1c_ level, 8.3% [1.9%] vs 7.8% [1.6%]; *P* < .001) (eTable 3 in Supplement 2). Based on the number of visits or calls documented, 0.5% of patients (n = 4) in the usual care group, 19.8% (n = 159) in the empowered care group, and 24.1% (n = 192) in the team-based empowered care group did not adhere to the protocol (Figure 1). The mean (SD) number of telephone visits in the empowered care group was 2.8 (1.1), and the mean (SD) number of telephone and/or clinic visits in the team-based empowered care group was 5.7 (1.6).

### Outcomes

**Figure 2.**
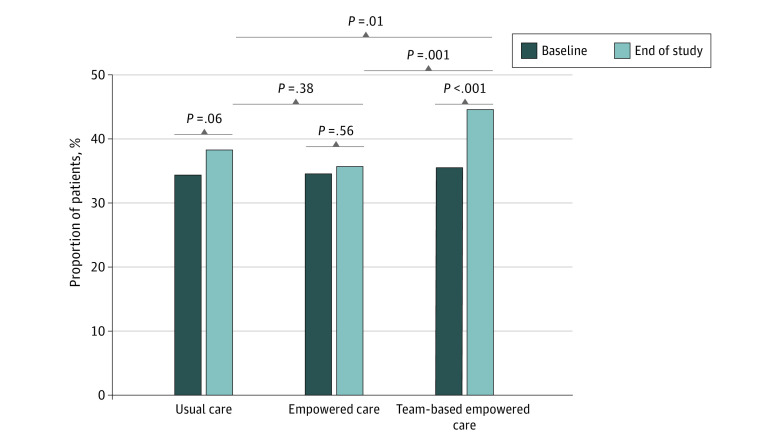
Changes in the Proportion of Patients Attaining at Least 3 Treatment Targets at Study End in the Intention-to-Treat Population The McNemar test was used for within-group and χ^2^ test for between-group comparisons of categorical variables. The within-group differences were 3.9% (95% CI, 0.0%-7.8%) in the usual care group, 1.3% (95% CI, −2.8% to 5.4%) in the empowered care group, and 9.1% (95% CI, 4.7%-13.5%) in the team-based empowered care group.

The team-based empowered care group (44.6%) had the highest proportion of patients who attained multiple treatment targets compared with those in the usual care (38.2%) or empowered care (35.7%) groups at month 12 (Figure 2). The within-group differences were 3.9% (95% CI, 0.0%-7.8%) in the usual care group, 1.3% (95% CI, −2.8% to 5.4%) in the empowered care group, and 9.1% (95% CI, 4.7%-13.5%) in the team-based empowered care group.

Patients in the team-based empowered care group compared with the usual care group (RR, 1.17; 95% CI, 1.00-1.37) and the empowered care group (RR, 1.25; 95% CI, 1.06-1.48) were more likely to attain multiple treatment targets under model 1 (adjusted for site). The team-based empowered care group had an RR of 1.20 (95% CI, 1.03-1.40) compared with the usual care group and an RR of 1.27 (95% CI, 1.07-1.49) compared with the empowered care group (Table 2) under model 2 (adjusted for site and baseline insulin use). Patients who attained multiple treatment targets were older, were predominantly men, received more intensified treatment (except for insulin), and showed better self-management at baseline compared with patients who did not attain multiple treatment targets (eTable 4 in Supplement 2).

**Table 2.** Comparison of Group Randomization Effect on Attainment of Multiple Treatment Targets^a^

Group	Model 1^b^		Model 2^c^	
	RR (95% CI)	*P* value	RR (95% CI)	*P* value
Team-based empowered care vs usual care^d^	1.17 (1.00-1.37)	.04	1.20 (1.03-1.40)	.02
Empowered care vs usual care^d^	0.94 (0.79-1.11)	.45	0.95 (0.80-1.12)	.54
Team-based empowered care vs empowered care^e^	1.25 (1.06-1.48)	.007	1.27 (1.07-1.49)	.005

Abbreviation: RR, risk ratio.

^a^ The number of patients attaining multiple treatment targets was 304 (38.2%) in the usual care group, 286 (35.7%) in the empowered care group, and 355 (44.6%) in the team-based empowered care group.

^b^ Model 1 was adjusted for site.

^c^ Model 2 involved model 1 plus insulin use at baseline.

^d^ Usual care was the reference.

^e^ Empowered care was the reference.

By month 12, the team-based empowered care group had greater reductions in mean (SD) HbA_1c_ level (−0.39% [1.76%]; *P* = .004) and LDL-cholesterol level (−0.14 [1.35] mmol/L; *P* = .001) compared with the usual care group (HbA_1c_ level, −0.18% [1.76%]; LDL-cholesterol level, 0.02 [1.11] mmol/L) and empowered care group (HbA_1c_ level, −0.15% [1.55%]; LDL-cholesterol level, 0.09 [1.47] mmol/L) (Table 3). Compared with the usual care (1.0% higher than baseline) and empowered care (1.3% lower than baseline) groups, more patients in the team-based empowered care group were prescribed lipid-lowering drugs and had greater attainment of an LDL-cholesterol level less than 1.8 mmol/L (7.8% higher than baseline; *P* < .001) at month 12 vs baseline (Table 3). More patients in the empowered care group (2.7% higher than baseline) and team-based empowered care group (7.3% higher than baseline) practiced self-management compared with patients in the usual care group (1.3% higher than baseline) (Table 3). Among all patients (n = 2393), 33 deaths (1.4%) occurred, and among patients without previous events (n = 1526), 187 (12.2%) had incident cardiovascular, kidney, and cancer events, with a similar distribution across the 3 groups (eTable 5 in Supplement 2). Compared with patients who did not attain multiple treatment targets, patients who did attain these targets had a lower incidence of cardiovascular, kidney, and cancer events, especially cardiovascular disease (8.4% [n = 51] vs 14.5% [n = 134]; *P* = .004) (eTable 6 in Supplement 2). Patients who did or did not attain multiple treatment targets had similar risks for cardiovascular, kidney, and cancer events after adjustment for age, sex, diabetes duration, site, and baseline target attainment in the intention-to-treat population (RR, 0.80; 95% CI, 0.55-1.18) (eTable 7 in Supplement 2) and per-protocol population (RR, 0.84; 95% CI, 0.52-1.34) (eTable 8 in Supplement 2).

**Table 3.** Changes in Cardiometabolic Risk Factors and Medication Use From Baseline to Month 12 by Group Randomization in the Intention-to-Treat Population

Variable	Mean (SD)									*P* value^a^
	Usual care group (n = 795)			Empowered care group (n = 802)			Team-based empowered care group (n = 796)			
	Baseline	Mo 12	Difference	Baseline	Mo 12	Difference	Baseline	Mo 12	Difference	
HbA_1c_ level, %	7.9 (1.7)	7.7 (1.7)	−0.18 (1.76)	7.8 (1.6)	7.7 (1.6)	−0.15 (1.55)	7.9 (1.7)	7.5 (1.8)	−0.39 (1.76)	.004
HbA_1c_ level, mmol/mol	62.4 (18.3)	60.5 (18.6)	−1.94 (19.21)	62.1 (17.2)	60.4 (17.5)	−1.67 (16.93)	63.0 (18.1)	58.8 (19.9)	−4.2 (19.2)	.004
Systolic BP, mm Hg	138.8 (19.5)	137.9 (20.4)	−0.88 (22.01)	138.5 (17.6)	137.6 (21.6)	−0.85 (22.2)	138.3 (18.5)	137.2 (21.2)	−1.14 (22.8)	.89
Diastolic BP, mm Hg	74.2 (11.1)	73.0 (13.1)	−1.19 (12.82)	74.4 (10.7)	73.3 (13.0)	−1.18 (12.6)	74.2 (11.6)	73.0 (13.6)	−1.22 (14.00)	.95
BMI	26.8 (4.5)	26.7 (4.7)	−0.06 (1.98)	27.1 (4.9)	26.9 (4.7)	−0.15 (2.47)	26.9 (4.8)	26.8 (4.9)	−0.07 (2.02)	.54
Total cholesterol level, mmol/L	4.4 (1.1)	4.4 (1.3)	0.02 (1.20)	4.4 (1.1)	4.4 (1.6)	0.04 (1.54)	4.3 (1.1)	4.2 (1.4)	−0.12 (1.36)	.046
Triglyceride level, mmol/L	1.9 (1.3)	1.9 (1.6)	0.02 (1.43)	1.9 (1.4)	1.9 (1.4)	−0.08 (1.34)	1.9 (1.3)	1.9 (1.8)	−0.01 (1.61)	.27
HDL-cholesterol level, mmol/L	1.2 (0.4)	1.2 (0.5)	0.004 (0.50)	1.2 (0.4)	1.2 (0.6)	0.03 (0.57)	1.2 (0.4)	1.2 (0.5)	−0.02 (0.50)	.13
LDL-cholesterol level, mmol/L	2.3 (0.9)	2.4 (1.2)	0.02 (1.11)	2.4 (1.1)	2.5 (1.5)	0.09 (1.47)	2.4 (1.2)	2.2 (1.3)	−0.14 (1.35)	.001
Urinary ACR, mg/mmol	58.6 (130.8)	79.6 (213.5)	21.1 (191.2)	61.2 (142.9)	71.7 (167.9)	10.49 (158.67)	68.2 (149.5)	72.3 (216.7)	4.12 (186.31)	.03
eGFR, mL/min/1.73m^2^	50.6 (16.5)	50.6 (22.7)	−0.04 (18.18)	49.0 (15.7)	48.1 (23.1)	−0.95 (19.39)	49.6 (16.8)	49.1 (23.6)	−0.53 (17.67)	.38
HbA_1c_ level <7.0% (<53 mmol/mol), %^b^	34.9	34.3	−0.6	32.4	32.9	0.5	30.8	40.3	9.5	<.001
BP <130/80 mm Hg, %^b^	28.7	30.9	2.2	26.5	32.1	5.6	28.9	30.6	1.7	.66
LDL-cholesterol level <1.8 mmol/L, %^b^	26.2	27.2	1.0	25.3	24.0	−1.3	28.2	36.0	7.8	<.001
Triglyceride level <1.7 mmol/L, %^b^	54.5	56.3	1.8	53.6	55.6	2.0	53.4	55.0	1.6	.92
RAAS inhibitors, %^b^	69.2	61.8	−7.4	68.7	63.6	−5.1	70.9	65.2	−5.7	.47
BP-lowering drugs, %^b^	84.0	76.5	−7.5	82.2	77.3	−4.9	84.9	79.1	−5.8	.56
Lipid-lowering drugs, %^b^	78.7	74.1	−4.6	76.6	74.8	−1.8	76.8	78.0	1.2	.04
Noninsulin glucose-lowering drugs, %^b^	84.0	81.9	−2.1	81.0	80.8	−0.2	82.8	81.5	−1.3	.83
Insulin, %^b^	43.6	42.9	−0.7	48.3	49.1	0.8	50.4	48.7	−1.7	.27
At least 2 self-care activities,%^b^^,^^c^	63.8	65.1	1.3	66.0	68.7	2.7	64.1	71.4	7.3	.02

Abbreviations: ACR, albumin to creatinine ratio; BMI, body mass index (calculated as weight in kilograms divided by height in meters squared); BP, blood pressure; eGFR, estimated glomerular filtration rate; HbA_1c_, glycated hemoglobin; HDL, high-density lipoprotein; LDL, low-density lipoprotein; RAAS, renin-angiotensin-aldosterone system.

SI conversion factor: to convert HbA_1c_ to the proportion of total hemoglobin, multiply by 0.01.

^a^ *P* values denote 1-way analysis of variance test statistic for 3 group comparisons of differences between baseline and month 12.

^b^ SD values were not available.

^c^ Self-care activities included self-monitoring of blood glucose at least once per week, physical activity at least 3 times per week, and adherence to a balanced diet in the past 3 months.

In the per-protocol analysis, all 3 groups had similar baseline profiles except for higher frequency of general obesity and lower insulin use in the usual care group (eTable 9 in Supplement 2). A greater proportion of patients in the team-based empowered care group attained multiple treatment targets at month 12 than patients in the empowered care group (41.7% vs 35.6%; *P* = .04) (eFigure in Supplement 2). Compared with patients in the usual care group, those in the team-based empowered care group (RR, 1.24; 95% CI, 1.03-1.49), but not those in the empowered care vs usual care group (RR, 0.95; 95% CI, 0.78-1.15), attained multiple treatment targets at month 12 (eTable 10 in Supplement 2). Among patients without previous events, the incidence of cardiovascular, kidney, and cancer events was similar across all 3 groups (eTable 11 in Supplement 2) and between patients who did or did not attain multiple treatment targets (eTable 12 in Supplement 2).

## Discussion

In this multinational RCT involving patients with DKD, randomization to the team-based empowered care group compared with the usual care group or empowered care group increased the likelihood of attaining multiple treatment targets by 17% to 27% (RR of 1.17 to 1.27). Both the team-based empowered care and empowered care groups showed better self-management, although nurse support alone in the empowered care group did not increase the likelihood of attaining multiple targets. There was also increased use of statins (lipid-lowering drugs) in the team-based empowered care group. In treating patients with complex needs, such as those with DKD, physicians and nurses can complement one another in minimizing multiple risk factors by providing continual structured care aimed at improving patient-clinician communication, patient self-management, and the timely use of organ-protective drugs.

Previous JADE studies found that less than 10% of patients with type 2 diabetes attained a composite target of an HbA_1c_ level less than 7.0% (53 mmol/mol), BP lower than 130/80 mm Hg, and LDL-cholesterol level less than 2.6 mmol/L, and fewer than 50% of patients were treated with organ-protective drugs.^12,13^ In the present RCT, 34.7% of patients attained at least 3 targets, with 70% to 80% of them treated with organ-protective drugs at baseline; this finding highlights the diversity of care standards within the same country, region, or location. These favorable profiles at baseline may have attenuated the effect size of the intervention. However, we were able to confirm that team-based empowered care increased the proportion of patients who attained multiple treatment targets by 9.1% compared with no change in the usual care or empowered care groups.

Compared with the usual care group or the empowered care group, the team-based empowered care group had greater reductions in HbA_1c_ and LDL-cholesterol levels and increased statin use, with higher proportions of patients attaining multiple targets. Because of the short follow-up time, low event rates, and high use of organ-protective drugs at baseline, there was no difference in clinical events between the 3 groups. However, patients who attained multiple targets had a lower rate of any clinical events than patients who did not attain multiple targets, extending the findings of the Steno-2 Study (Intensified Multifactorial Intervention in Patients With Type 2 Diabetes and Microalbuminuria),^28^ the SURE Study,^24^ and the J-DOIT3 trial (Japan Diabetes Optimal Integrated Treatment Study for 3 Major Risk Factors of Cardiovascular Diseases)^29^ to patients with DKD.

Although experts advocate the use of team-based integrated care to prevent end-stage kidney disease, there are few studies to inform its implementation in clinical practice.^1,30^ The multifunctional and multilingual JADE web portal is a prototype for using information and communication technology to fill the implementation gaps. The built-in protocols provide guidance to nurses for systematic data collection and for generating a simple-to-read personalized report that includes risk categories, trends for modifiable risk factors (BP, lipids, HbA_1c_, and body weight), future risks for major events, and tailored decision support.^31^

The JADE personalized report aims to promote physician and patient communication. Although the physician report focuses on care gaps, the patient report focuses on self-management and treatment adherence. By performing structured assessments every 12 to 18 months in line with international recommendations,^32^ a physician-nurse team can establish a register to benchmark performance and recall defaulters. In a previous JADE RCT of 20 834 patients with type 2 diabetes in 8 countries or regions, the JADE technology–assisted intervention group was more likely to attain at least 2 treatment targets (HbA_1c_ level <7.0% [53 mmol/mol], BP <130/80 mm Hg, or LDL-cholesterol level <2.6 mmol/L) after 12 months, with larger effects (odds ratios) in low- and middle-income countries compared with high-income countries (50% vs 20%).^31^

In the present RCT, only the team-based empowered care group had improved control of multiple risk factors, likely because of the complex medical needs of these patients, although the favorable effect on self-management in the empowered care group was encouraging. Patients who attained multiple treatment targets had better risk factors and self-care as well as lower insulin use at baseline compared with patients who did not attain multiple targets. These findings lend support to the importance of behavior in influencing glycemic control.^33^ Patient default is a major challenge in the management of chronic and silent conditions, such as diabetes. In a multicenter RCT including Chinese patients with type 2 diabetes but without DKD, the provision of the JADE personalized report reduced all cardiometabolic risk factors and additional nurse contacts improved self-management and decreased the default rate.^34^ In the present study, all patients were given detailed written information regarding the rationale and purpose of the trial, and the high-risk nature of DKD was emphasized during the consent process. This approach might explain the high return rate in all 3 groups at month 12 (87.4% in the usual care group, 86.9% in the empowered care group, and 85.3% in the team-based empowered care group), with those who did not return showing worse risk factor control at baseline than those who returned. Taken together, the results of this trial concord with those of a meta-analysis regarding the benefits of multicomponent, data-driven integrated care,^15^ which recommended early referral to an interdisciplinary team, including specialist support, to improve outcomes in patients with complex needs such as DKD.^30^

### Strengths and Limitations

This RCT has several strengths. In this academic-led study by a charitable organization, we adopted a pragmatic randomized design to evaluate the feasibility, acceptability, and effects of using technology and nonphysician personnel to address unmet needs in DKD, especially in low-resource settings. Despite limited funds to support extensive on-site monitoring, we had put in place appropriate strategies to optimize fidelity. We conducted online data monitoring using the JADE web portal and issuing regular reminders to the study sites. Despite the feasibility and benefits of this care model, it is important to align institutional support, capacity building, and incentives to increase its value. Given the efficacy of multicomponent integrated care,^15^ self-management,^33^ and use of organ-protective drugs in clinical trial settings,^35^ the results of this RCT have provided an implementation prototype to payers, policy makers, and health care practitioners for translating evidence to practice.^36,37^

This RCT also has several limitations. First, apart from the volunteer bias of patients, investigators, and study sites, the inclusion of patients with mild DKD may limit the generalizability of the results to those with more advanced DKD. Nevertheless, the implementation of structured care early in the clinical course can provide considerable public health impact.^16^ Second, the study population had relatively good control of risk factors at baseline, with approximately 70% of them treated with RAAS inhibitors and approximately 77% treated with lipid-lowering drugs. This finding might lead to healthy user bias and limit the use of empowered care and team-based empowered care to further improve care. Third, despite the high return rate for end-of-study reassessment, 24.1% in the team-based empowered care group and 19.8% in the empowered care group did not adhere to intervention procedures. Practical reasons (eg, taking leave from work) aside, in Asia, many patients still perceive physicians as the main caregivers and may not appreciate the role of nurse educators. Given that team-based empowered care can increase the likelihood of attaining multiple targets, additional strategies are needed to improve protocol adherence and recall defaulters. Understanding the cognitive and psychological factors and perspectives of patients can improve the sustainability of the team-based empowered care program.^21^ Fourth, in the per-protocol analysis, exclusion of patients who received additional phone calls in the team-based empowered care and empowered care groups and those with extra visits in the usual care group might have attenuated the results. We relied on data entry into the JADE web portal as evidence of contacts that might have taken place without documentation. Fifth, few study sites had centralized electronic medical records, and thus we based all clinical events on patient recalls and hospitalization notes. Thus, ascertainment bias might have led to the overreporting of clinical events in the empowered care and team-based empowered care groups given their regular contacts with the investigators, and to the underreporting in the usual care group, which had only baseline and end-of-study assessments. In addition, the pragmatic study design focused on care processes and was underpowered to estimate the effects of interventions on clinical events.

## Conclusions

This RCT found that using a physician-nurse team to implement multicomponent, data-driven integrated care, with the assistance of information technology (JADE web portal in this trial), can improve communication between patients and health care practitioners as well as result in the reduction of multiple risk factors, attainment of multiple treatment targets, and empowerment among high-risk patients with DKD.

## Notes

Supplement 1.
Trial Protocol

Supplement 2. **eTable 1.** Distribution of Randomized Patients by Study Sites and Regions **eTable 2.** Definition of Clinical Events **eTable 3.** Comparison of Baseline Clinical Characteristics Between Patients Who Did or Did Not Return for End-of-Study Assessment **eTable 4.** Comparison of Baseline Clinical Characteristics by Target Attainment at 12 Months in the Intention-to-Treat Population **eTable 5.** Number of Patients With Incident Clinical Events by Group Randomization in the Intention-to-Treat Population **eTable 6.** Number of Patients With Incident Non-Fatal Clinical Events by Target Attainment at 12 Months in the Intention-to-Treat Population **eTable 7.** Poisson Regression to Show the Association Between Incident Non-Fatal Clinical Events and Target Attainment at 12 Months in the Intention-to-Treat Population **eTable 8.** Poisson Regression to Show the Association Between Incident Non-Fatal Clinical Events and Target Attainment at 12 Months in the Per-Protocol Population **eTable 9.** Baseline Clinical Characteristics of Patients Who Adhered to the Study Protocol and Returned for End-of-Study Assessment by Group Randomization in the Per-Protocol Population **eTable 10.** Poisson Regression to Show the Association of Assignment to Team-Based Empowered Care Compared With Empowered Care and Usual Care on Attainment to Multiple Treatment Targets at 12 Months in the Per-Protocol Population **eTable 11.** Number of Patients With Incident Clinical Events by Group Randomization at 12 Months in the Per-Protocol Population **eTable 12.** Number of Patients With Incident Non-Fatal Clinical Events by Target Attainment at 12 Months in the Per-Protocol Population **eFigure.** Changes in the Proportion of Patients Attaining at Least 3 Treatment Targets at 12 Months in the Per-Protocol Population

Supplement 3.
Data Sharing Statement
