# Supplementary material for: Effect of a Web-Based Management Guide on Risk Factors in Patients With Type 2 Diabetes and Diabetic Kidney Disease: A JADE Randomized Clinical Trial
Source: JAMA Netw Open. 2022 Mar 25;5(3):e223862. doi: 10.1001/jamanetworkopen.2022.3862 (PMC8956973; doi:10.1001/jamanetworkopen.2022.3862)
Supplement: Supplement 1. — Trial Protocol [file jamanetwopen-e223862-s001.pdf]

USING A DIABETIC KIDNEY DISEASE (DKD) REGISTRY TO TREAT TO MULTIPLE TARGETS (TMT) (DKD-TMT)

Proposed by Professor Juliana CN Chan
Professor of Medicine and Therapeutics
The Chinese University of Hong Kong (CUHK), Prince of Wales Hospital (PWH)
Co-director, CUHK-PWH International Diabetes Federation Centre of Education (IDFCE)
Chief Executive Officer, Asia Diabetes Foundation (ADF)
Hong Kong SAR CHINA
Contact: jchan@cuhk.edu.hk Tel: 852 9189 2868 (mobile) or 2637 6624 (ADF)
Protocol Date: January 9, 2015

CONFIDENTIAL

Rationale

There is now a pandemic of diabetes affecting all areas and races, including the young and the old. Diabetes reduces life expectancy by 6 years especially in young people and increases vascular, renal, cancer and all-cause death by 1.3-3 folds [1]. Compared to their white counterparts, non-Caucasians are at higher risk for developing diabetic kidney disease (DKD) [2] which substantially increases risk of cardiovascular diseases (CVD) and all-cause death[3].

In clinical trial settings, control of blood pressure [4], blood glucose [5] and blood lipids[6] as well as inhibition of the renin angiotensin system (RAS) has been shown to reduce albuminuria [7], slow rate of decline in renal function [8] and reduce risk of CVD and end stage renal disease (ESRD) [9,10]. In a post hoc analysis of the Treating to New Target Trial, treatment with 10 mg and 80 mg atorvastatin was associated with increased eGFR, more so in the high dose group [8]. Yet, in most national and international surveys, less than 30% of patients were treated to target and/or received life-saving drugs such as statins and RAS inhibitors with an even lower percentage of patients treated to multiple targets [11-13]. Apart from clinical inertia[14], 30-50% of patients treated with chronic medications for silent conditions such as diabetes and hypertension were non-compliant and unable to benefit from these life-saving drugs [15,16].

Since 1995, using a trio team consisting of a doctor, nurse and data manager, the CUHK-PWH Diabetes Care and Research Team has established a diabetes registry using predefined protocols to document risk factors, complications, drug use and clinical outcomes. This ongoing registry comprising 10,000 patients has provided enormous insights into the natural history of diabetes and impact of appropriate drug use on clinical outcomes [17-21]. Using a team approach, augmented by care protocols with periodic reminders, we were able to substantially increase the rate of attaining multiple treatment targets and reduce the risk of major endpoints including CVD, ESRD and death in these high risk subjects [22-24]. In support of these findings, in a recent systematic analysis, quality improvement initiatives with particular focus on empowering patients to improve self care were associated with significant improvement in risk factor control [25].

In 2007, we designed a web-based internet program (Joint Asia Diabetes Evaluation (JADE)

Program) incorporating templates for data collection, validated risk engines and decision support to enable doctors to establish his/her diabetes registry for quality assurance and ongoing evaluation. The collection and organization of these data into an easy-to-read format which displays individualized risk profile, trends of risk factor control with decision support has reduced clinical inertia and empowered patients with better self care. Upon reassessment at 1 year after enrolment, 30% of patients had meaningful reduction in risk factors (e.g. 0.5% for A1c, 30% for LDL-C, 10 mmHg for systolic BP, 3% for body weight) and improved rate of attainment of treatment targets [26-28].

Hypothesis

- Patient empowerment and team-based care augmented by the JADE program with features of risk stratification and decision support improves multiple risk factor control in Asian type 2 diabetic patients with chronic kidney disease
- Attaining multiple treatment targets reduces all diabetes-related endpoints in these high risk patients

Objectives

- To use the JADE program to set up a diabetic kidney disease (DKD) registry and document control of risk factors and care standards in real practice in Asian type 2 diabetic patients
- To use the JADE program with features of risk stratification, feedback, recommendations and decision support to empower doctors and patients to make informed decisions
- To use a team approach augmented by the JADE program to treat to multiple targets and reduce all diabetes related clinical outcomes

Inclusion criteria

- Type 2 diabetic patients with serum creatinine above 30% upper limit of normal of the laboratory range and/or estimated glomerular filtration rate (eGFR)< 65ml/min/$1.73m^2$; or type 2 diabetic patients with both eGFR between 60 to 90ml/min/$1.73m^2$ and urine albumin creatinine ratio (ACR) ≥ 25 mg/mmol.
- Willingness to return for regular follow up visits

Exclusion criteria

- Inability to give informed consent
- Life threatening condition with reduced life expectancy
- Patients on dialysis or eGFR<15 ml/min/1.73^2

Study design

This will be a multicentre randomized translational program to compare the effects of usual care (UC) versus empowered care (EC) versus team-based, empowered care (TEC) on risk factor control and clinical outcomes in 3000 patients with DKD in Asian countries.

CONFIDENTIAL

Setting

All patients will be recruited from high volume clinics in Asia led by diabetologists or nephrologists or cardiologists or internists. Each centre will consecutively recruit 300 patients with DKD and randomize them to UC (n=100), EC (n=100) or TEC (n=100) groups. After giving written informed consent, they will be randomized using sealed envelope, opened by an independent personnel, containing randomly generated computer codes for group assignment. All patients will undergo comprehensive assessments including examination of eye and feet as well as collection of blood and urine samples at baseline and 1 year later in accordance to international recommendations [29]. Each patient will be followed up at least for 1 year and assuming half year of set up and patient recruitment, the project will last for 1.5 years. At the end of 1.5 years, the clinical outcomes of all enrolled patients will be ascertained by telephone contact within 1 month of the due date of repeat assessment of the last recruited patient.

Intervention

Usual care (UC) group

After undergoing comprehensive assessment, all patients will receive UC in accordance to the practice of the health institution and return at 12 months for repeat assessment

Empowered care (EC) group

After undergoing comprehensive assessment, all patients will be given a JADE summary report with personalized risk prediction, treatment targets and decision support with explanation from the doctor and nurse (Appendices 1 and 2). In addition to receiving UC in accordance to the practice of the health institution, the nurse will telephone the patient 3-monthly to remind them to adhere to treatment, provide support and empower them to discuss with their doctors about their treatment needs and any concerns. No reports will be given to patients as part of these follow up contacts in the EC group. All patients will return at 1 year for repeat assessment.

Team-based, empowered care (TEC) group

Patients randomized to the TEC group will be followed up by a doctor-nurse team at least 3 monthly to achieve multiple targets recommended as A1c<7%, BP<130/80 mmHg, LDL-C<1.8 mmol/l, triglyceride<1.7 mmol/l and persistence with RAS inhibitors, but tailored to patients' risk profile. Between follow-up visits, a nurse will telephone the patients. The patients will also be given a JADE follow up report 3-monthly and return at 12 month for repeat comprehensive assessment.

Group	N	Month 0	Month 1.5	Month 3	Month 4.5	Month 6	Month 7.5	Month 9	Month 12
UC	100	CA/N							CA/N
EC	100	CA/N/D		N-Tel FU		N-Tel FU		N-Tel FU	CA/N/D
TEC	100	CA/N/D	N-Tel FU	N/D/TMT	N-Tel FU	N/D/TMT	N-Tel FU	N/D/TMT	CA/N/D

CA=comprehensive assessment, N=nurse, D=doctor TMT=treat to multiple targets

CONFIDENTIAL

Comprehensive assessment and data collection

All investigators and nurses will attend a 1-day training workshop on the 'what, why and how' of structured care and patient empowerment using a team approach, how to use the internet-based JADE portal [27 28 26] and how to explain the results to patients with appropriate follow up actions. The JADE portal provides structured templates to guide care providers to collect demographic, clinical, biochemical, quality of life and drug usage data in accordance to standards for diabetes care recommended by the International Diabetes Federation (IDF)[29]. The portal also enables doctors to establish a registry at their own clinic as a quality improvement initiative.

Questionnaires

A set of self-administered questionnaires (Appendix 5) for assessing quality of life and psychological distress will be given to patients in all 3 groups to complete when they undergo comprehensive assessments at baseline and at 12 months.

(i) Quality of Life (QOL)

QOL will be assessed with the EQ-5D-3L, the World Health Organization Quality Of Life-BREF (WHOQOL-BREF), and a Time Trade-Off (TTO) question.

- The EQ-5D-3L assesses 5 dimensions of quality of life, namely, mobility, self-care, usual activities, pain/discomfort and anxiety/depression. Each dimension has 3 levels: no problems, some problems, extreme problems[30]. The whole instrument consists of 2 pages, including a 5-item descriptive system, and an EQ visual analogue scale (EQ VAS) for a person to indicate their best imaginable health state.
- The WHOQOL-BREF[31] is a shortened and validated version of the WHOQOL, which is a cross-culturally applicable QOL assessment. It comprises 26 items and measures the broad domains of physical health, psychological health, social relationships, and environment.
- The TTO question[32] consists of a horizontal line which is marked and labelled 0,1,2,…10 years. The TTO question reads as follows: "Imagine that you are told that you have 10 years left to live. In connection with this you are told that you can choose to live these 10 years in your current health state or that you can choose to give up some life years to live for a shorter period in full health. Indicate with a cross on the line the number of years in full health that you think is equal value to 10 years in your current health state.". The TTO value is obtained by dividing the response to the above question by 10. The TTO question has been used in multiple health-related investigations[33-35].

(ii) Psychological Distress

Psychological distress will be assessed with the Patient Health Questionnaire-9 (PHQ-9) and the Depression Anxiety Stress Scale (DASS-21).

- PHQ-9 is a 9-item scale that assesses depression severity and can be used for depression screening in primary care settings[36]. Its introduction reads: "Over the last 2 weeks, how often have you been bothered by any of the following problems?". A sample item is

CONFIDENTIAL

"Little interest or pleasure in doing things." Response options range from 0 ("Not at all") to 3 ("Nearly every day"). Higher scale scores indicate more severe depression. This scale has been validated and shown satisfactory reliability in research with Asian samples[37].

- DASS-21 is a 21-item scale that assesses depression, anxiety, and stress. Participants are asked to read each of the 21 items and rate on a scale of 0 ("do not apply to me at all") to 3 ("applied to me very much, or most of the time"). A sample item is "I couldn't seem to experience any positive feeling at all". Higher scale scores indicate greater emotional distress. This scale has been validated and shown satisfactory reliability in research with Asian samples[38].

Follow up action after comprehensive assessment

Patients randomized to the UC group will undergo comprehensive assessment but will not be given any report after comprehensive assessments. It will be up to their attending doctor to explain the results and take actions in accordance to their usual practice. For patients randomized to the EC and TEC groups, after comprehensive assessments the patients will be issued a JADE summary report listing their risk category:

- 4=cardiovascular disease and/or ESRD and/or eGFR<15ml/min/$1.73m^2$
- 3=≥3 risk parameters and/or eGFR 15-60 ml/min/$1.73m^2$ and/or high risk scores for future cardiovascular-renal events
- 2=2 risk parameters and/or moderate risk for future cardiovascular-renal events and
- 1=1 or no risk factor and no CKD and low risk for future events

The report will also display the 5-year probability of future events based on validated equations using data collected during comprehensive assessment[28]. In the EC group, no JADE follow up report will be given to patients. In the TEC group only, the nurse will issue a JADE follow up report 3-monthly to inform the patients regarding their trends of risk factor control with decision support.

Research subject protection

This study protocol is in compliance with the Declaration of Helsinki. It is also in compliance with ICH-GCP.

Outcome measures

- Primary outcome[22]
  - attainment of at least 3 treatment targets defined as
    - A1c<7%
    - BP<130/80 mmHg
    - LDL-C<1.8 mmol/L
    - Triglyceride<1.7 mmol/L
    - Use of RAS inhibitors between the 3 groups

CONFIDENTIAL

- Secondary outcomes[22]
  - Incidence of all-diabetes related endpoints (vascular, cancer, non-vascular non-cancer and all-cause death) between patients who attain at least 3 targets versus those who do not
- Other outcomes
  - amongst the UC, EC and TEC groups
    - Absolute and % changes in A1c, lipids, BP and body weight
    - Absolute and % changes in eGFR and albuminuria
    - Use of medications including statins, RAS inhibitors and insulin
  - between patients treated to multiple targets and those without
    - Incidence of ESRD and all-cause death
    - Incidence of all CV events including stroke, acute myocardial infarction, heart failure, acute coronary syndrome, all-cause death
    - Incidence of all-site cancer and all-cause death
    - Use of medications including statins, RAS inhibitors and insulin
- Cost-effectiveness and quality of life analysis

Structure and implementation

The JADE Program was developed and administered by the Asia Diabetes Foundation (ADF, www.adf.org.hk), a charitable organization registered under the Hong Kong Government and governed by the Chinese University of Hong Kong Foundation. In this investigator-initiated project, the ADF will provide the portal support and project management including portal training, database management and analysis. All data are collected on an anonymous basis which will contribute to a DKD Registry steered by international and regional experts with representatives from the sponsor for analysis, reporting and communication purposes. A committee consisting of nephrologists, diabetologists, cardiologists, epidemiologists and biostatisticians will meet regularly to ascertain the outcome measures based on narratives and supporting data. Members of the ADF team will visit the sites at least once a year to review the progress of the study while the JADE portal will be used to monitor the speed of patient recruitment and quality of data entry, followed by teleconference as and when required. All investigators and steering committee members will meet at least 3 times during the 1.5-year study period, i.e. at commencement, half way and completion. At least 1 nurse and 1 doctor responsible for study implementation will visit Hong Kong to attend a 1-day training workshop to familiarize themselves with the JADE portal and the workflow of implementing structured and team-based care.

Sample size estimation

Most surveys in developing areas (Asia, East Europe and South America) show that only 20-30% of patients were treated with statins or RAS inhibitors or attained 1 of the 3 ABC goals (A1c<7%, BP<130/80 mmHg and LDL-C<2.6 mmol/l) and only 3-10% attained multiple treatment

CONFIDENTIAL

goals[11,13]. Based on our experiences using the JADE Program which has enrolled more than 30,000 patients since its launch in 2007, the organization, presentation and communication of data in an easy-to-read format has empowered patients to improve compliance with better physical and psychological health[39].

The power analysis and sample size estimation (Appendices 3 and 4) was conducted using PASS 11 (NCSS, LLC. Kaysville, Utah, USA. www.ncss.com). We estimated the rates of attainment of multiple treatment targets to be 10%, 20% and 30% in the UC, EC and TEC groups respectively. A sample size of 1000 patients in each of the 3 groups will have >95% power to test the primary hypothesis using a 2-sided likelihood ratio test and taking into consideration of multiple comparisons (type I error set at 1%). We further estimated that patients in the UC group will at least have an event rate of 5% per year[40], and EC group will have a 37% risk reduction (i.e., HR=0.63)[18,22]. A sample size of 2000 in total (1000 in the UC group and 1000 in the EC group) will have 80% power to confirm the effect size at an alpha of 0.05 assuming a yearly attrition rate of 20% in each group, half year for enrolment and 1 year for follow up and no switch in intervention between groups. For the comparison of the TEC with the UC group, we take multiple comparisons into consideration, i.e., type I error at 2.5%. A sample size of 2000 in total (1000 in the UC group and 1000 in the TEC group) will have 80% power to confirm the effect size of 50% risk reduction (HR=0.50) [18,22] assuming an attrition rate of 20% in each group, half year for enrolment and 1 year for follow up and no switch in intervention between groups. Thus, a total of 3000 patients, 1000 in each group, is needed to achieve the effect sizes with 80% power and taking into consideration of multiple comparisons.

Statistical analysis

All analyses will be performed using the Statistical Program for Social Sciences (SPSS). All data will be expressed as mean±SD or median (interquartile range) as appropriate. Skewed data will be logarithmically transformed. Analysis of variance, student’s t test and Fisher’s exact-test will be used for comparisons amongst the UC, EC and TEC groups, as appropriate. The Kaplan Miere estimator will be used to demonstrate the cumulative incidence of first diabetes-related event while Cox model with adjustment for covariables at baseline and during follow-up will be used to check their contributions to the observed risk reductions. A p-value <0.05 (2-sided) will be considered significant. Number needed to treat (NNT) for prevention of an event by the EC and TEC group will be calculated separately using survival functions.

Significance

Results from this pragmatic program will define the epidemiology of DKD in Asian population and the effects of empowerment and team-based care, augmented by information technology, in reducing adverse clinical outcomes and its cost-effectiveness.

Data ownership and publication

CONFIDENTIAL

While each site will be able to use the JADE portal to establish its own DKD registry after the publication of the main paper, the anonymized dataset will be owned by the ADF and the sponsor that funds the investigators to establish the registry. The ADF will coordinate the data analysis under the guidance of a steering committee. All members of the steering committee will be co-authors of the paper and publish the main results on behalf of the JADE-DKD Study Group. Authorship and authorship order will be determined according to the International Committee of Medical Journal Editors (ICMJE) Uniform Requirements for Manuscripts Submitted to Medical Journals (http://www.icmje.org/#author).

References

1. Seshasai SR, Kaptoge S, Thompson A, et al. Diabetes mellitus, fasting glucose, and risk of cause-specific death. N Engl J Med 2011;364:829-41.
2. Karter A, Ferrara A, Liu J, Moffet H, Ackerson L, Selby J. Ethnic disparities in diabetic complications in an insured population. Journal of American Medical Association 2002;287:2519-27.
3. Matsushita K, van der Velde M, Astor BC, et al. Association of estimated glomerular filtration rate and albuminuria with all-cause and cardiovascular mortality in general population cohorts: a collaborative meta-analysis. Lancet 2010;375:2073-81.
4. Bakris GL, Williams M, Dworkin L, et al. Preserving renal function in adults with hypertension and diabetes: a consensus approach. National Kidney Foundation Hypertension and Diabetes Executive Committees Working Group. American Journal of Kidney Diseases 2000;36:646-61.
5. Patel A, MacMahon S, Chalmers J, et al. Intensive blood glucose control and vascular outcomes in patients with type 2 diabetes. N Engl J Med 2008;358:2560-72.
6. Colhoun HM, Betteridge DJ, Durrington PN, et al. Effects of atorvastatin on kidney outcomes and cardiovascular disease in patients with diabetes: an analysis from the Collaborative Atorvastatin Diabetes Study (CARDS). Am J Kidney Dis 2009;54:810-9.
7. Parving HH, Lehnert H, Brochner-Mortensen J, et al. The effect of irbesartan on the development of diabetic nephropathy in patients with type 2 diabetes. New England Journal of Medicine 2001;345:870-8.
8. Shepherd J, Kastelein JJ, Bittner V, et al. Effect of intensive lipid lowering with atorvastatin on renal function in patients with coronary heart disease: the Treating to New Targets (TNT) study. Clin J Am Soc Nephrol 2007;2:1131-9.
9. Baigent C, Landray MJ, Reith C, et al. The effects of lowering LDL cholesterol with simvastatin plus ezetimibe in patients with chronic kidney disease (Study of Heart and Renal Protection): a randomised placebo-controlled trial. Lancet;377:2181-92.
10. Brenner BM, Cooper ME, de Zeeuw D, et al. Effects of losartan on renal and cardiovascular outcomes in patients with type 2 diabetes and nephropathy. N Engl J Med 2001;345:861-9.
11. Chan JCN, Gagliardino JJ, Baik SH, et al. Multi-faceted Determinants For Achieving

CONFIDENTIAL

Glycaemic Control: The International Diabetes Management Practice Study (IDMPS). Diabetes Care 2009;32:227-33.

12. Chan JC, Chan SP, Deerochanawong C, et al. Diabetic dyslipidaemia in Asian populations in the Western Pacific Region: What we know and don't know. Diabetes Res Clin Pract.

13. Bhatt D, Steg P, Ohman E, et al. International prevalence, recognition, and treatment of cardiovascular risk factors in outpatients with atherothrombosis. Journal of American Medical Association 2006;11:180-9.

14. Grant RW, Buse JB, Meigs JB. Quality of diabetes care in U.S. academic medical centers: low rates of medical regimen change. Diabetes Care 2005;28:337-442.

15. Ho PM, Rumsfeld JS, Masoudi FA, et al. Effect of medication nonadherence on hospitalization and mortality among patients with diabetes mellitus. Arch Intern Med 2006;166:1836-41.

16. Wu JY, Leung WY, Chang S, et al. Effectiveness of telephone counselling by a pharmacist in reducing mortality in patients receiving polypharmacy: randomised controlled trial. Bmj 2006;333:522.

17. Chan JC, So WY, Ma RCW, Tong P, Wong R, Yang XL. The complexity of both vascular and non-vascular complications of diabetes: The Hong Kong Diabetes Registry. Curr Cardiovasc Risk Rep 2011;5:230-9.

18. Kong AP, Yang X, Ko GT, et al. Effects of treatment targets on subsequent cardiovascular events in Chinese patients with type 2 diabetes. Diabetes Care 2007;30:953-9.

19. Luk AO, Yang X, Ma RC, et al. Association of statin use and development of renal dysfunction in type 2 diabetes--the Hong Kong Diabetes Registry. Diabetes Res Clin Pract;88:227-33.

20. Ting RZ, Yang X, Yu LW, et al. Lipid control and use of lipid-regulating drugs for prevention of cardiovascular events in Chinese type 2 diabetic patients: a prospective cohort study. Cardiovasc Diabetol;9:77.

21. So WY, Chan N, Tong PCY, et al. Effect of RAAS inhibition on survival and renal outcomes in 3737 Chinese Type 2 diabetic patients. Hypertension 2004;44:294-9.

22. Chan JCN, So WY, Yeung CY, et al. The SURE Study: Effects of Structured versus Usual care on Renal Endpoint in Type 2 diabetes: A randomized multi-centre translational study Diabetes Care 2009;32:977-82.

23. Chan JC, Malik V, Jia W, et al. Diabetes in Asia: epidemiology, risk factors, and pathophysiology. Jama 2009;301:2129-40.

24. So WY, Chan JC. The role of the multidisciplinary team. In: Holt R, Cockram S, Flyvbjerg A, Goldstein B, eds. Textbook of Diabetes Blackwell; 2010:969-83.

25. Tricco AC, Ivers NM, Grimshaw JM, et al. Effectiveness of quality improvement strategies on the management of diabetes: a systematic review and meta-analysis. Lancet;379:2252-61.

26. Ko GT, So WY, Tong PC, et al. From design to implementation--the Joint Asia Diabetes Evaluation (JADE) program: a descriptive report of an electronic web-based diabetes management program. BMC Med Inform Decis Mak 2010;10:26.

CONFIDENTIAL

27. So WY, Raboca J, Sobrepena L, et al. Comprehensive risk assessments of diabetic patients from seven Asian countries: The Joint Asia Diabetes Evaluation (JADE) program*. J Diabetes 2011;3:109-18.
28. Chan J, So W, Ko G, et al. The Joint Asia Diabetes Evaluation (JADE) Program: a web-based program to translate evidence to clinical practice in Type 2 diabetes. Diabet Med 2009;26:693-9.
29. IDF Clinical Guidelines Task Force. Global guideline for type 2 diabetes: recommendations for standard, comprehensive, and minimal care. . Diabet Med 2006;23:579-93.
30. Rabin R, de Charro F. EQ-5D: a measure of health status from the EuroQol Group. Annals of Medicine 2001;33:337-43.
31. Harper A, Power M, Grp W. Development of the World Health Organization WHOQOL-BREF quality of life assessment. Psychol Med 1998;28:551-8.
32. Burstrom K, Johannesson M, Diderichsen F. A comparison of individual and social time trade-off values for health states in the general population. Health Policy 2006;76:359-70.
33. Bardage C, Isacson D, Ring L, Bingefors K. A Swedish population-based study on the relationship between the SF-36 and health utilities to measure health in hypertension. Blood Pressure 2003;12:203-10.
34. Lundberg L, Johannesson M, Isacson DGL, Borgquist L. The relationship between health-state utilities and the SF-12 in a general population. Medical Decision Making 1999;19:128-40.
35. Lundberg L, Johannesson M, Isacson DGL, Borgquist L. Health-state utilities in a general population in relation to age, gender and socioeconomic factors. European Journal of Public Health 1999;9:211-7.
36. Kroenke K, Spitzer RL, Williams JBW. The PHQ-9 - Validity of a brief depression severity measure. Journal of General Internal Medicine 2001;16:606-13.
37. Zhang YY, Ting R, Lam M, et al. Measuring depressive symptoms using the Patient Health Questionnaire-9 in Hong Kong Chinese subjects with type 2 diabetes. Journal of Affective Disorders 2013;151:660-6.
38. Chan JCN, Sui Y, Oldenburg B, et al. Effects of Telephone-Based Peer Support in Type 2 Diabetic Patients Receiving Integrated Care. JAMA Internal Medicine In press.
39. Chan JC, Sui Y, Chung HHY, et al. A Randomized Study To Evaluate Peer Support In Type 2 Diabetic Patients Receiving Structured Care. 72th American Diabetes Association Scientific Symposium Philadelphia, USA2012.
40. Luk AOY, So WY, Ma RCW, et al. Metabolic Syndrome Predicts New Onset of Chronic Kidney Disease in 5,829 Patients With Type 2 Diabetes A 5-year prospective analysis of the Hong Kong Diabetes Registry. Diabetes Care 2008;31:2357-61.

CONFIDENTIAL

Appendix 1. **Sample of JADE Summary Report (in different languages for patients' report)**

The summary report of risk categorization, complications and risk parameters are based on the results of the latest or last available COMPREHENSIVE ASSESSMENT (CA). Occurrence of new complications or risk factors will not be reflected until next CA visit.

Care Level as of 11-Sep-2013 **3** For details of care level, please refer to Risk Stratification.

High risk for future vascular complications. Low eGFR (<60) and/or having ≥ 3 risk factors.

Date of most recent contact: 11-Sep-2013 **Disease duration:** 4 years
Gender: Male **Age:** 56 **Ethnicity:** Chinese **Occupation:** Retired
Drug Allergies and Other Notes:
NKDA

Getting the patient to target

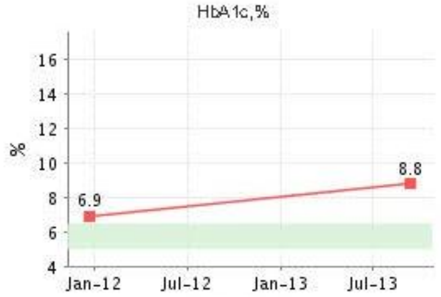

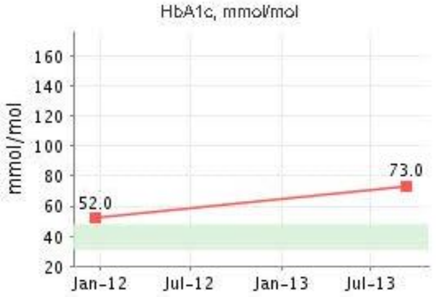

HbA_{1c}

Recommendations

- Consider referring patient to diabetes nurse specialist for review.
- Poor glycaemic control.
- Reinforce compliance and SBGM, review BG lowering regimen, may need insulin, early FU is indicated.
- Check HbA_{1c} once every 3 months

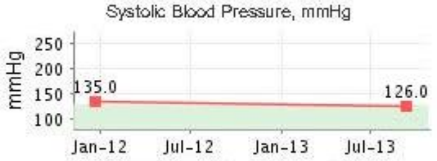

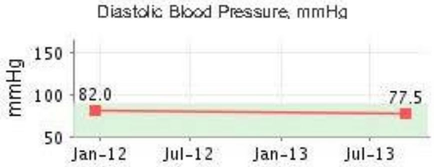

BP

Recommendations

- BP on target

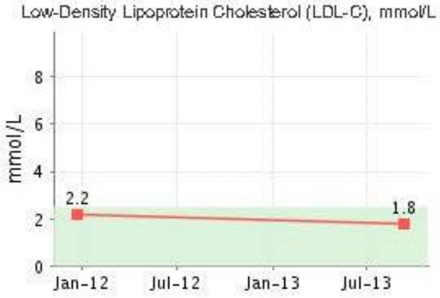

Low-Density Lipoprotein Cholesterol (LDL-C)

Recommendations

- Optimal LDL-C control
- Check LDL-C at least once every year.

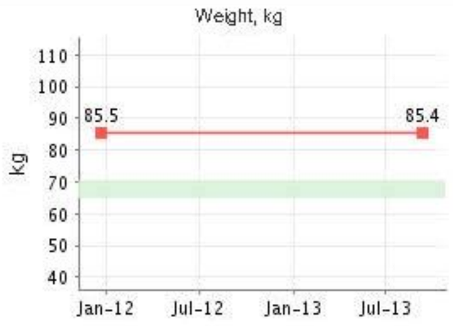

Body Weight

Recommendations

- Consider referring patient to dietician for review.
- Reinforce Exercise Target
- Reinforce Diet Compliance
- Reinforce importance of normal body weight.
- Obese.
- Central obesity.
- Look out for obesity related complications e.g. obstructive sleep apnoea (OSA).
- Exclude other causes for weight gain such as fluid retention and secondary causes of obesity e.g. Cushing's syndrome or hypothyroidism. Expect weight gain with improved BG control, e.g. start of insulin, SU or glitazones

Cardiovascular-Renal Complications

Patient does not have any cardiovascular-renal complications.

Stratification Parameters

- Albuminuria
- Dyslipidaemia: Hypertriglyceridaemia and/or low HDL
- Hypertension
- Obesity
- Poor Glycaemic Control

5 Year Probability (%)

The 5-year probability of complications is based on published results derived from the Hong Kong Diabetes Registry and may not be applicable to all ethnic groups or patients living outside Hong Kong.

The primary aim of listing these probabilities based on the annual comprehensive assesment is to enable doctors and patients to make informed decisions and choices regarding their diabetes management.

The listed treatment targets and testing procedures are for recommendations only and should be individually tailored. Both doctors and patients are recommended to set and work towards a realistic goal to improve risk factor control and reduce risk for compliciations.

For more information, please visit the following websites: www.idf.org, www.diabetes.org, www.yckdac.hkido.cuhk.edu.hk, www.adf.org.hk.

The ESRD risk equation was revised on 19th May 2008.

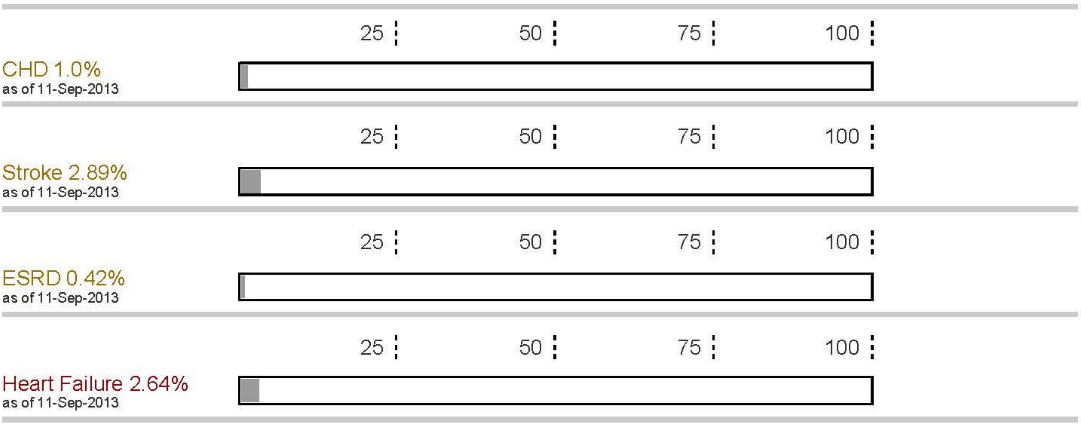

Note:- Different risk parameters may have different weightings, thus within the same risk level, there can be a wide range of 5 year probability risks of events.

The 5-year probability of complications are based on data available at the CURRENT visit and will only be generated if required data are available.

Doctor's and Nurse's Comments :

Risk category	4	3	2	1
Cardiovascular disease and/or renal failure	Yes	No	No	No
Renal impairment (estimated glomerular filtration rate ml/min/1.73m2)	Severe (<15 or dialysis)	Moderate (15-60)	Mild (60-90)	Normal (>=90)
Risk factors	NA	At least 3	2	0-1
Future risk for complications based on risk scores	Very High	High	Moderate	Low
Recommended number of medical review per year	Doctors and patients are encouraged to discuss and formulate a mutually agreeable management plan. Frequent contacts with doctors or other care professionals are often needed at diagnosis for education and treatment adjustment or if control worsens. Once stable, most patients can be reviewed every 2-4 months. At least 6-12 monthly medical reviews are recommended for low risk patients due to possible silent deterioration.			
Recommended interval for comprehensive assessments	Every 12-24 months especially for patients with irregular contacts with health care professionals.			

Prescription Details

Generic drug name	Dosage	Frequency	Route	Start Date	Status
Metformin	1 g	(bd (twice a day))	Oral	01-Sep-2011	Previous
Sitagliptin	100 mg	(qd (daily))	Oral	01-Sep-2011	Previous
Caduet 10/20 mg tab	1 tab	(qd (daily)) om	Oral		New
Hyzaar Forte tab	1 tab (100/25mg)	(qd (daily)) om	Oral		New

Next Visit

Agreed Date for Next Contact: _____ - _____

Doctor / Clinic

Name: ____________________

Signature/Stamp:

References

Yang X et al. *Diabetes Care* 30:65-70, 2007; Yang X et al. *Am J Cardiol* 101:596-601, 2008; Yang X et al. *Diabetologia* 50:1348-1350, 2007; Yang X et al. *Cardiovasc Diabetol* 7:9, 2008; Yang X et al. *Arch Intern Med* 168:451-457, 2008; Chan JC et al. *Diabetes Care* 32:977-982, 2009; Chan J et al. *Diabet Med* 26:693-699, 2009; Ko GT et al. *BMC Med Inform Decis Mak;*10:26, 2010; So WY et al. *J Diabetes;*3:109-118, 2011

Disclaimer

Asia Diabetes Foundation Limited (referred to herein as "ADF") hereby expressly disclaims any and all liability as follows: any and all data, information and result including without limitation in connection with JADE doctor summaries and patient reports, whether medically based or otherwise, which is provided under the JADE Program shall be used for reference purpose only. The JADE Program is not intended in any shape or form to be used, construed, interpreted or applied as a substitute for professional medical care or advice which shall be provided by a doctor, physician or other qualified medical/health care professionals. Users of the JADE Program (including without limitations to patients and family members of patients), who receive, believe, rely or make decisions regarding their health or medical care based on risk predictions provided by the JADE Program do so at their own risk. Users are reminded to seek professional medical care or advice provided by a doctor, physician or other qualified medical/health care professionals after receiving results or reports from the JADE Program. Neither the author(s) of the above papers nor ADF including without limitations to staff, employees and consultants (if any) are liable or incur any duty of care for any decisions and/or consequences arising from or in relation to the assessment and/or interpretation of the results from the JADE Program. ADF is a charitable institution incorporated in Hong Kong and a subsidiary of the Chinese University of Hong Kong Foundation with a mission to achieve positive impacts for the better understanding and care of diabetes and other chronic diseases. All rights including without limitation intellectual property belong to ADF. No part of any publication may be reproduced or transmitted in any form without the prior written permission of ADF. In the event of any dispute or discrepancy between the English version and the translation of other languages of this Disclaimer, the English version shall prevail.

Appendix 2. **Sample of JADE Comprehensive Assessment Report**

Joint Asia Diabetes Evaluation (JADE) - Comprehensive Assessment

ADF Patient Code: HKM195703087796 Date(dd/mmm/yyyy): 11-Sep-2013
Patient Last Name: ______ Patient First Name: ______
Attending Doctor: ______ Completed by(Nurse): ______ Last Modified By: YCK1

Personal and family history

Age:	56
Gender:	Male
Occupation:	Retired
Job nature:	Others
Highest education attained:	Middle School (>6 & 11 years)
Smoking:	Never
Number of cigarettes/day:	None
Years of smoking:	0.0
Use of alcohol:	Never
Alcohol units per week:	
Diabetic father:	No
Diabetic mother:	Yes
Diabetic siblings:	No
Renal disease affecting 1st degree relatives:	No
Premature CVD affecting 1st degree relatives:	No
Cancers affecting 1st degree relatives:	No

Details of family history:
Mother (stroke at age 70+)

Diabetes related complications, illnesses and symptoms

Disease duration: 4 Years Year of diagnosis: 2009 Definite Type 1 diabetes: No

	Diagnosis	Year of 1st event	Year of last event	No of new events since last FU/CA	Total no of events
Coronary heart disease:	No				
Coronary arterial intervention:	No			0	0
Myocardial	No			0	0
Cardiac failure:	No			0	0

Remarks on AHE (All Heart Events):

	Diagnosis	Year of 1st event	Year of last event	No of new events since last FU/CA	Total no of events
Stroke with full recovery:	No			0	0
Stroke with permanent residual	No			0	0

Remarks on stroke :

LL - lower limb; PVD - peripheral vascular disease

	Diagnosis	Year of event
Right LL amputation:	No	
RLL Non-traumatic amputation:	No	
Left LL amputation:	No	
LLL Non-traumatic amputation:	No	
Treatment for PVD:	No	
Renal transplantation:	No	
Dialysis:	No	

Treatment summary

	Diagnosis		
History of Hypertension:	Yes		
On BP lowering drugs:	Yes	Start year:	2011
On Lipid regulating drugs:	Yes	Start year:	2011
Anti-diabetic treatment:	OAD only		
Insulin treatment start year:			
No of insulin injections:			
Total daily dose(units):			

Significant symptoms

Calf pain on walking:	No	Impotence:	Yes
Abnormal sensation in LL:	No	Coughing:	No
Shortness of breath:	Yes	Edema:	No
Chest pain:	No		

Diabetes education | Follow-up | Self care

Regular follow-up (At least once in last 12 months):	Yes
Type of follow-up:	[Community based clinic, Self medication]
Type of clinic:	[Private]
Education by dietitian :	No
Education by podiatrist:	No
Education by DM nurses:	No
Frequency of hypoglycemia in last 3 months:	None
Nature of hypoglycemic attack:	
Number of severe hypo attacks requiring attention in last 3 months:	0
Self monitoring of glucose control:	None
Self monitoring in last 3 months:	None
Physical activity in last 3 months:	No regular activity
Adherence to balanced diet in last 3 months:	Occasional
Ever forgotten or self adjusted your medication:	No
Level of medication adherence (0-100):	100

Remarks by educators:

Cancers and other medical conditions

Major operations (with year):
Nil

Other illnesses (with year):
Fatty liver (2000's)

Site of malignancy	Diagnosis	Year of 1st event	Year of last event	No of new events since last FU/CA	Total no of events
Lung:				0	0
Breast:				0	0
Gynecological:				0	0
Hepato-biliary:				0	0
Prostate:				0	0
Colo-rectal:				0	0
Upper GI:				0	0
NPC:				0	0
Other malignancy:				0	0

Remarks on other sites cancer

Active treatment for cancer: No
Known Hepatitis B carrier: Yes

Drug Allergy (details)

NKDA

Details of symptoms

C/O SOB when walking upslope; C/O pain on dorsum of feet while walking

Joint Asia Diabetes Evaluation (JADE) -Comprehensive Assessment

ADF Patient Code: HKM195703087796 Date(dd/mmm/yyyy): 11-Sep-2013
Patient Last Name: ______ Patient First Name: ______
Attending Doctor: ______ Completed by(Nurse): ______ Last Modified By: YCK1

Physical examination

Height:	1.68 m	Weight:	85.4 kg
BMI:	30.26 kg/m^2		
Waist:	106 cm	Hip:	105 cm
Waist hip ratio (WHR):	1.01		
Systolic BP(Right):	130 mmHg	(Left):	122 mmHg
Diastolic BP(Right):	81 mmHg	(Left):	74 mmHg
Pulse:	72 b/min		
Urine RBC:	-ve		
Urine ketone:			
Urine protein:	trace		

Laboratory results

Plasma sodium:	138 mmol/L	Plasma potassium:	3.9 mmol/L
Plasma urea:	6.9 mmol/L	Plasma creatinine:	91 µmol/L
eGFR:	97.964 mL/min per 1.73m^2	Plasma uric acid:	0.38 mmol/L
Plasma calcium:	2.64 mmol/L	Plasma phosphate:	0.91 mmol/L
Urine albumin:	49.5 mg/L	Urine creatinine:	19.5 mmol/L
Urine ACR (lab value):	2.5 mg/mmol		
Haematocrit:	0.42 L/L	Haemoglobin:	13.8 g/dL
Platelet:	248 10^9/L	White cell count:	12.1 10^9/L
ALP:	84 IU/L	ALT:	52 IU/L
Albumin:	45 g/L	Bilirubin:	12 µmol/L
Total cholesterol:	3.5 mmol/L	Triglyceride:	2 mmol/L
HDL-C:	0.8 mmol/L	LDL-C:	1.8 mmol/L
HbA1c:	8.8 %	Fasting PG:	9.5 mmol/L
Random PG:		2 hour PG:	

Action Items

Agreed date of next contact:
Referral to DM nurse:
Referral to dietitian:
Referral to podiatrist:
Referral to other specialists:
Others:

Blood test:
Urine test:
Special investigations:
Details of other investigations:

Quality of Life and Comments

1. Mobility	Some problems in walking about
2. Self-Care	No problems with self-care
3. Usual Activities (e.g. work, leisure activities)	Some problems with performing my usual activities
4. Pain/Discomfort	Moderate pain or discomfort
5. Anxiety/Depression	Not anxious or depressed
State of health:(best=100, worst=0):	50
State of happiness:(happy=100,unhappy=0):	80
Total Score for PHQ-9:	1
Total Score for DASS- 21:	

Reasons for suboptimal risk factor control:

Other reasons:

Doctor's and nurse's comments:
Refer to attached letter and JADE report.

Eye and Foot Examination

	(R)	(L)
Visual acuity (VA) (corrected with glasses or pinhole)	20/16	20/16
If VA >20/200:		
History of glaucoma:	No	No
History of cataract surgery:	No	No
History of laser treatment:	No	No
History of retinal surgery:	No	No
Fundus photo taken:	Yes	Yes
Retina seen by doctor:	Yes	Yes
Cataract seen by doctor:	No	No
Laser scars (seen by doctor):	No	No
DMR (seen by doctor):	No	No
Maculopathy:	No	No
Non-proliferative retinopathy:	No	No
Pre-proliferative retinopathy:	No	No
Proliferative retinopathy:	No	No
Advanced eye disease:	No	No
PVD (doctor diagnosed):	No	No
Sensory neuropathy (doctor diagnosed):	No	No
Diminished touch (by monofilament):	No	No
Vibration (by graduated tuning fork):	8/8	8/8
Diminished pulse (Dr's palpation):		
Abnormal wave (Doppler):		
Ankle systolic BP (Doppler):	135 mmHg	125 mmHg
Ankle:brachial ratio (ABR):	1.038	1.025
Acute foot ulcer or gangrene:	No	No
Foot deformities:		[None]

Skin abnormalities: [Dry skin, Fungal infection]

	(R)	(L)
PVD (derived term):	No	No
Sensory neuropathy (derived term):		
Foot at risk (derived term):	No	

Remarks:
Non-active tinea pedis on Lt foot; No eye FU

Special investigations

C peptide:		hsCRP:	
Fasting insulin:		Serum B12:	
HOMA%IR:		HOMA-Beta%:	

Abnormal CXR:
Abnormal CXR details:

Abnormal ECG: Yes
ECG abnormalities: [Others]
ECG other findings:
First degree heart block

Abnormal stress test:
Type of stress test:
Stress test details:

Abnormal cardiac imaging:
Type of cardiac imaging:
Carotid IMT date:

	(R)	(L)
Cartoid mean:	mm	mm
Carotid max:	mm	mm

Carotid plaque:
Abnormal carotid IMT:
Carotid IMT details:

Ultrasound scan date:
Kidney abnormalities:
Fatty liver:
Details:

Other investigations:

Appendix 3. **Sample size estimation for primary endpoint (treatment to multiple targets)**

8/12/2012 3:32:21 PM 1

Two Independent Proportions (Null Case) Power Analysis

Numeric Results of Tests Based on the Difference: P1 - P2
H0: P1-P2=0. H1: P1-P2=D1<>0. Test Statistic: Likelihood Ratio test

Power	Sample Size Grp 1 N1	Sample Size Grp 2 N2	Prop|H1 Grp 1 or Trtmnt P1	Prop Grp 2 or Control P2	Diff if H0 D0	Diff if H1 D1	Target Alpha	Actual Alpha	Beta
1.0000	500	500	0.1000	0.3000	0.0000	-0.2000	0.0100		0.0000
1.0000	550	550	0.1000	0.3000	0.0000	-0.2000	0.0100		0.0000
1.0000	600	600	0.1000	0.3000	0.0000	-0.2000	0.0100		0.0000
1.0000	650	650	0.1000	0.3000	0.0000	-0.2000	0.0100		0.0000
1.0000	700	700	0.1000	0.3000	0.0000	-0.2000	0.0100		0.0000
1.0000	750	750	0.1000	0.3000	0.0000	-0.2000	0.0100		0.0000
1.0000	800	800	0.1000	0.3000	0.0000	-0.2000	0.0100		0.0000

Note: exact results based on the binomial were only calculated when both N1 and N2 were less than 100.

References

Chow, S.C.; Shao, J.; Wang, H. 2003. Sample Size Calculations in Clinical Research. Marcel Dekker. New York.
D'Agostino, R.B., Chase, W., Belanger, A. 1988.'The Appropriateness of Some Common Procedures for Testing the Equality of Two Independent Binomial Populations', The American Statistician, August 1988, Volume 42 Number 3, pages 198-202.
Fleiss, J. L., Levin, B., Paik, M.C. 2003. Statistical Methods for Rates and Proportions. Third Edition. John Wiley & Sons. New York.
Lachin, John M. 2000. Biostatistical Methods. John Wiley & Sons. New York.
Machin, D., Campbell, M., Fayers, P., and Pinol, A. 1997. Sample Size Tables for Clinical Studies, 2nd Edition. Blackwell Science. Malden, Mass.

Report Definitions

'Power' is the probability of rejecting a false null hypothesis. It should be close to one.
'N1 and N2' are the sizes of the samples drawn from the corresponding populations.
'P1' is the proportion for group one under H1. This is the treatment or experimental group.
'P2' is the proportion for group two. This is the standard, reference, or control group
'Target Alpha' is the probability of rejecting a true null hypothesis that was desired.
'Actual Alpha' is the value of alpha that is actually achieved.
'Beta' is the probability of accepting a false null hypothesis.

Summary Statements

Group sample sizes of 500 in group one and 500 in group two achieve 100% power to detect a difference between the group proportions of -0.2000. The proportion in group one (the treatment group) is assumed to be 0.3000 under the null hypothesis and 0.1000 under the alternative hypothesis. The proportion in group two (the control group) is 0.3000. The test statistic used is the two-sided Likelihood Ratio test. The significance level of the test was targeted at 0.0100. The significance level actually achieved by this design is NA.

8/12/2012 3:32:22 PM 2

Two Independent Proportions (Null Case) Power Analysis

Chart Section

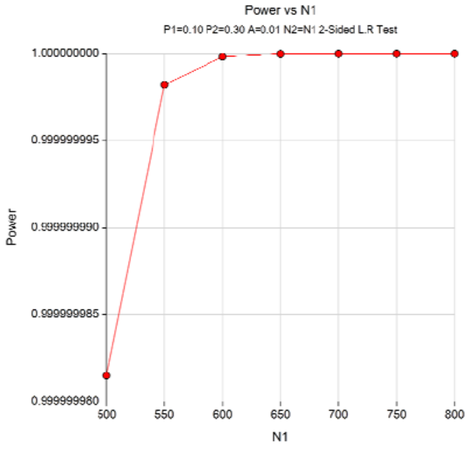

Two Independent Proportions (Null Case) Power Analysis

Numeric Results of Tests Based on the Difference: P1 - P2
H0: P1-P2=0. H1: P1-P2=D1<>0. Test Statistic: Likelihood Ratio test

Power	Sample Size Grp 1 N1	Sample Size Grp 2 N2	Prop|H1 Grp 1 or Trtmnt P1	Prop Grp 2 or Control P2	Diff if H0 D0	Diff if H1 D1	Target Alpha	Actual Alpha	Beta
0.8606	500	500	0.2000	0.3000	0.0000	-0.1000	0.0100		0.1394
0.8966	550	550	0.2000	0.3000	0.0000	-0.1000	0.0100		0.1034
0.9242	600	600	0.2000	0.3000	0.0000	-0.1000	0.0100		0.0758
0.9450	650	650	0.2000	0.3000	0.0000	-0.1000	0.0100		0.0550
0.9605	700	700	0.2000	0.3000	0.0000	-0.1000	0.0100		0.0395
0.9719	750	750	0.2000	0.3000	0.0000	-0.1000	0.0100		0.0281
0.9801	800	800	0.2000	0.3000	0.0000	-0.1000	0.0100		0.0199

Note: exact results based on the binomial were only calculated when both N1 and N2 were less than 100.

References

Chow, S.C.; Shao, J.; Wang, H. 2003. Sample Size Calculations in Clinical Research. Marcel Dekker. New York.

D'Agostino, R.B., Chase, W., Belanger, A. 1988.'The Appropriateness of Some Common Procedures for Testing the Equality of Two Independent Binomial Populations', The American Statistician, August 1988, Volume 42 Number 3, pages 198-202.

Fleiss, J. L., Levin, B., Paik, M.C. 2003. Statistical Methods for Rates and Proportions. Third Edition. John Wiley & Sons. New York.

Lachin, John M. 2000. Biostatistical Methods. John Wiley & Sons. New York.

Machin, D., Campbell, M., Fayers, P., and Pinol, A. 1997. Sample Size Tables for Clinical Studies, 2nd Edition. Blackwell Science. Malden, Mass.

Report Definitions

'Power' is the probability of rejecting a false null hypothesis. It should be close to one.
'N1 and N2' are the sizes of the samples drawn from the corresponding populations.
'P1' is the proportion for group one under H1. This is the treatment or experimental group.
'P2' is the proportion for group two. This is the standard, reference, or control group
'Target Alpha' is the probability of rejecting a true null hypothesis that was desired.
'Actual Alpha' is the value of alpha that is actually achieved.
'Beta' is the probability of accepting a false null hypothesis.

Summary Statements

Group sample sizes of 500 in group one and 500 in group two achieve 86% power to detect a difference between the group proportions of -0.1000. The proportion in group one (the treatment group) is assumed to be 0.3000 under the null hypothesis and 0.2000 under the alternative hypothesis. The proportion in group two (the control group) is 0.3000. The test statistic used is the two-sided Likelihood Ratio test. The significance level of the test was targeted at 0.0100. The significance level actually achieved by this design is NA.

Two Independent Proportions (Null Case) Power Analysis

Chart Section

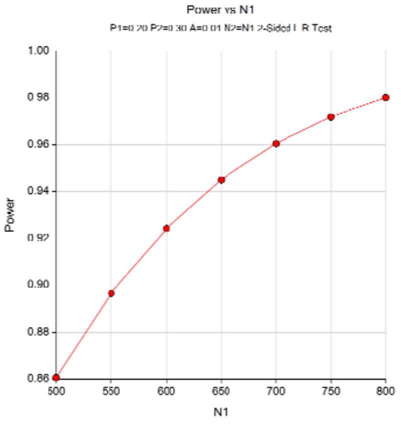

8/12/2012 3:30:31 PM 1

Two Independent Proportions (Null Case) Power Analysis

Numeric Results of Tests Based on the Difference: P1 - P2
H0: P1-P2=0. H1: P1-P2=D1<>0. Test Statistic: Likelihood Ratio test

Power	Sample Size Grp 1 N1	Sample Size Grp 2 N2	Prop|H1 Grp 1 or Trtmnt P1	Prop Grp 2 or Control P2	Diff if H0 D0	Diff if H1 D1	Target Alpha	Actual Alpha	Beta
0.9693	500	500	0.1000	0.2000	0.0000	-0.1000	0.0100		0.0307
0.9816	550	550	0.1000	0.2000	0.0000	-0.1000	0.0100		0.0184
0.9892	600	600	0.1000	0.2000	0.0000	-0.1000	0.0100		0.0108
0.9937	650	650	0.1000	0.2000	0.0000	-0.1000	0.0100		0.0063
0.9964	700	700	0.1000	0.2000	0.0000	-0.1000	0.0100		0.0036
0.9980	750	750	0.1000	0.2000	0.0000	-0.1000	0.0100		0.0020
0.9989	800	800	0.1000	0.2000	0.0000	-0.1000	0.0100		0.0011

Note: exact results based on the binomial were only calculated when both N1 and N2 were less than 100.

References

Chow, S.C.; Shao, J.; Wang, H. 2003. Sample Size Calculations in Clinical Research. Marcel Dekker. New York.
D'Agostino, R.B., Chase, W., Belanger, A. 1988.'The Appropriateness of Some Common Procedures for Testing the Equality of Two Independent Binomial Populations', The American Statistician, August 1988, Volume 42 Number 3, pages 198-202.
Fleiss, J. L., Levin, B., Paik, M.C. 2003. Statistical Methods for Rates and Proportions. Third Edition. John Wiley & Sons. New York.
Lachin, John M. 2000. Biostatistical Methods. John Wiley & Sons. New York.
Machin, D., Campbell, M., Fayers, P., and Pinol, A. 1997. Sample Size Tables for Clinical Studies, 2nd Edition. Blackwell Science. Malden, Mass.

Report Definitions

'Power' is the probability of rejecting a false null hypothesis. It should be close to one.
'N1 and N2' are the sizes of the samples drawn from the corresponding populations.
'P1' is the proportion for group one under H1. This is the treatment or experimental group.
'P2' is the proportion for group two. This is the standard, reference, or control group
'Target Alpha' is the probability of rejecting a true null hypothesis that was desired.
'Actual Alpha' is the value of alpha that is actually achieved.
'Beta' is the probability of accepting a false null hypothesis.

Summary Statements

Group sample sizes of 500 in group one and 500 in group two achieve 97% power to detect a difference between the group proportions of -0.1000. The proportion in group one (the treatment group) is assumed to be 0.2000 under the null hypothesis and 0.1000 under the alternative hypothesis. The proportion in group two (the control group) is 0.2000. The test statistic used is the two-sided Likelihood Ratio test. The significance level of the test was targeted at 0.0100. The significance level actually achieved by this design is NA.

8/12/2012 3:30:31 PM 2

Two Independent Proportions (Null Case) Power Analysis

Chart Section

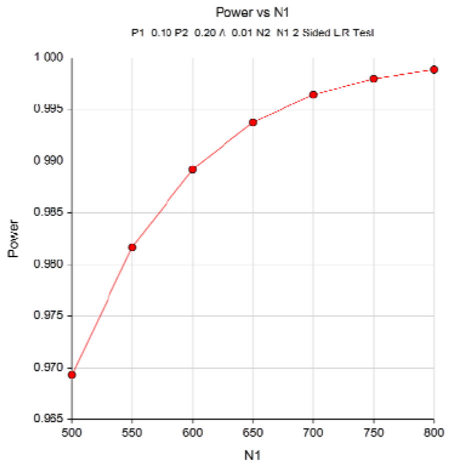

Appendix 4. **Sample size estimation for secondary endpoint (all diabetes-related endpoints)**

8/12/2012 4:38:47 PM 1

Logrank Test Power Analysis

Numeric Results in Terms of Sample Size when the Test is Two-Sided

Power	N1	N2	N	Haz Ratio (HR)	Ctrl Haz Rate (h1)	Trt Haz Rate (h2)	Acc-rual Pat'n	Acc-rual Time/ Total Time	Ctrl Loss	Trt Loss	Ctrl to Trt	Trt to Ctrl	Alpha	Beta
0.9993	1000	1000	2000	0.4000	0.0500	0.0200	Equal	1 / 3	0.2000	0.2000	0.0000	0.0000	0.0500	0.0007
0.9985	1000	1000	2000	0.4200	0.0500	0.0210	Equal	1 / 3	0.2000	0.2000	0.0000	0.0000	0.0500	0.0015
0.9968	1000	1000	2000	0.4400	0.0500	0.0220	Equal	1 / 3	0.2000	0.2000	0.0000	0.0000	0.0500	0.0032
0.9937	1000	1000	2000	0.4600	0.0500	0.0230	Equal	1 / 3	0.2000	0.2000	0.0000	0.0000	0.0500	0.0063
0.9886	1000	1000	2000	0.4800	0.0500	0.0240	Equal	1 / 3	0.2000	0.2000	0.0000	0.0000	0.0500	0.0114
0.9805	1000	1000	2000	0.5000	0.0500	0.0250	Equal	1 / 3	0.2000	0.2000	0.0000	0.0000	0.0500	0.0195
0.9686	1000	1000	2000	0.5200	0.0500	0.0260	Equal	1 / 3	0.2000	0.2000	0.0000	0.0000	0.0500	0.0314
0.9517	1000	1000	2000	0.5400	0.0500	0.0270	Equal	1 / 3	0.2000	0.2000	0.0000	0.0000	0.0500	0.0483
0.9290	1000	1000	2000	0.5600	0.0500	0.0280	Equal	1 / 3	0.2000	0.2000	0.0000	0.0000	0.0500	0.0710
0.8999	1000	1000	2000	0.5800	0.0500	0.0290	Equal	1 / 3	0.2000	0.2000	0.0000	0.0000	0.0500	0.1001
0.8639	1000	1000	2000	0.6000	0.0500	0.0300	Equal	1 / 3	0.2000	0.2000	0.0000	0.0000	0.0500	0.1361
0.8433	1000	1000	2000	0.6100	0.0500	0.0305	Equal	1 / 3	0.2000	0.2000	0.0000	0.0000	0.0500	0.1567
0.8210	1000	1000	2000	0.6200	0.0500	0.0310	Equal	1 / 3	0.2000	0.2000	0.0000	0.0000	0.0500	0.1790
0.7971	1000	1000	2000	0.6300	0.0500	0.0315	Equal	1 / 3	0.2000	0.2000	0.0000	0.0000	0.0500	0.2029
0.7716	1000	1000	2000	0.6400	0.0500	0.0320	Equal	1 / 3	0.2000	0.2000	0.0000	0.0000	0.0500	0.2284
0.7167	1000	1000	2000	0.6600	0.0500	0.0330	Equal	1 / 3	0.2000	0.2000	0.0000	0.0000	0.0500	0.2833
0.6574	1000	1000	2000	0.6800	0.0500	0.0340	Equal	1 / 3	0.2000	0.2000	0.0000	0.0000	0.0500	0.3426
0.5951	1000	1000	2000	0.7000	0.0500	0.0350	Equal	1 / 3	0.2000	0.2000	0.0000	0.0000	0.0500	0.4049

References

Lakatos, Edward. 1988. 'Sample Sizes Based on the Log-Rank Statistic in Complex Clinical Trials', Biometrics, Volume 44, March, pages 229-241.

Lakatos, Edward. 2002. 'Designing Complex Group Sequential Survival Trials', Statistics in Medicine, Volume 21, pages 1969-1989.

Report Definitions

Power is the probability of rejecting a false null hypothesis. Power should be close to one.
N1|N2|N are the sample sizes of the control group, treatment group, and both groups, respectively.
Hazard Ratio (HR) is treatment group's hazard rate divided by the control group's hazard rate.
Hazard Rate is the instantaneous failure rate. Its scale is events per time period.
Accrual Time is the number of time periods (years or months) during which accrual takes place.
Total Time is the total number of time periods in the study. Follow-up time = (Total Time) - (Accrual Time).
Ctrl Loss is the proportion of the control group that is lost (drop out) during a single time period (year or month).
Trt Loss is the proportion of the treatment group that is lost (drop out) during a single time period (year or month).
Ctrl to Trt (drop in) is the proportion of the control group that switch to a group with a hazard rate equal to the treatment group.
Trt to Ctrl (noncompliance) is the proportion of the treatment group that switch to a group with a hazard rate equal to the control group.
Alpha is the probability of rejecting a true null hypothesis. It should be small.
Beta is the probability of accepting a false null hypothesis. It should be small.

8/12/2012 4:38:48 PM 2

Logrank Test Power Analysis

Numeric Results in Terms of Events when the Test is Two-Sided

Power	Ctrl Evts (E1)	Trt Evts (E2)	Total Evts (E)	Haz Ratio (HR)	Ctrl Haz Rate (h1)	Trt Haz Rate (h2)	Acc-rual Pat'n	Acc-rual Time/ Total Time	Ctrl Loss	Trt Loss	Ctrl to Trt	Trt to Ctrl	Alpha	Beta
0.9993	90.3	37.4	127.7	0.4000	0.0500	0.0200	Equal	1 / 3	0.2000	0.2000	0.0000	0.0000	0.0500	0.0007
0.9985	90.3	39.2	129.5	0.4200	0.0500	0.0210	Equal	1 / 3	0.2000	0.2000	0.0000	0.0000	0.0500	0.0015
0.9968	90.3	41.0	131.3	0.4400	0.0500	0.0220	Equal	1 / 3	0.2000	0.2000	0.0000	0.0000	0.0500	0.0032
0.9937	90.3	42.8	133.1	0.4600	0.0500	0.0230	Equal	1 / 3	0.2000	0.2000	0.0000	0.0000	0.0500	0.0063
0.9886	90.3	44.6	134.9	0.4800	0.0500	0.0240	Equal	1 / 3	0.2000	0.2000	0.0000	0.0000	0.0500	0.0114
0.9805	90.3	46.4	136.7	0.5000	0.0500	0.0250	Equal	1 / 3	0.2000	0.2000	0.0000	0.0000	0.0500	0.0195
0.9686	90.3	48.2	138.5	0.5200	0.0500	0.0260	Equal	1 / 3	0.2000	0.2000	0.0000	0.0000	0.0500	0.0314
0.9517	90.3	50.0	140.3	0.5400	0.0500	0.0270	Equal	1 / 3	0.2000	0.2000	0.0000	0.0000	0.0500	0.0483
0.9290	90.3	51.8	142.1	0.5600	0.0500	0.0280	Equal	1 / 3	0.2000	0.2000	0.0000	0.0000	0.0500	0.0710
0.8999	90.3	53.6	143.9	0.5800	0.0500	0.0290	Equal	1 / 3	0.2000	0.2000	0.0000	0.0000	0.0500	0.1001
0.8639	90.3	55.4	145.7	0.6000	0.0500	0.0300	Equal	1 / 3	0.2000	0.2000	0.0000	0.0000	0.0500	0.1361
0.8433	90.3	56.3	146.6	0.6100	0.0500	0.0305	Equal	1 / 3	0.2000	0.2000	0.0000	0.0000	0.0500	0.1567
0.8210	90.3	57.2	147.5	0.6200	0.0500	0.0310	Equal	1 / 3	0.2000	0.2000	0.0000	0.0000	0.0500	0.1790
0.7971	90.3	58.1	148.4	0.6300	0.0500	0.0315	Equal	1 / 3	0.2000	0.2000	0.0000	0.0000	0.0500	0.2029
0.7716	90.3	59.0	149.3	0.6400	0.0500	0.0320	Equal	1 / 3	0.2000	0.2000	0.0000	0.0000	0.0500	0.2284
0.7167	90.3	60.7	151.1	0.6600	0.0500	0.0330	Equal	1 / 3	0.2000	0.2000	0.0000	0.0000	0.0500	0.2833
0.6574	90.3	62.5	152.8	0.6800	0.0500	0.0340	Equal	1 / 3	0.2000	0.2000	0.0000	0.0000	0.0500	0.3426
0.5951	90.3	64.3	154.6	0.7000	0.0500	0.0350	Equal	1 / 3	0.2000	0.2000	0.0000	0.0000	0.0500	0.4049

Summary Statements

A two-sided logrank test with an overall sample size of 2000 subjects (1000 in the control group and 1000 in the treatment group) achieves 99.9% power at a 0.050 significance level to detect a hazard ratio of 00.4000 when the control group hazard rate is a hazard ratio of 00.0500. The study lasts for 3 time periods of which subject accrual (entry) occurs in the first time period. The proportion dropping out of the control group is 0.2000. The proportion dropping out of the treatment group is 0.2000. The proportion switching from the control group to another group with a hazard rate equal to the treatment group is 0.0000. The proportion switching from the treatment group to another group with a hazard rate equal to the control group is 0.0000.

8/12/2012 4:38:48 PM 3

Logrank Test Power Analysis

Chart Section

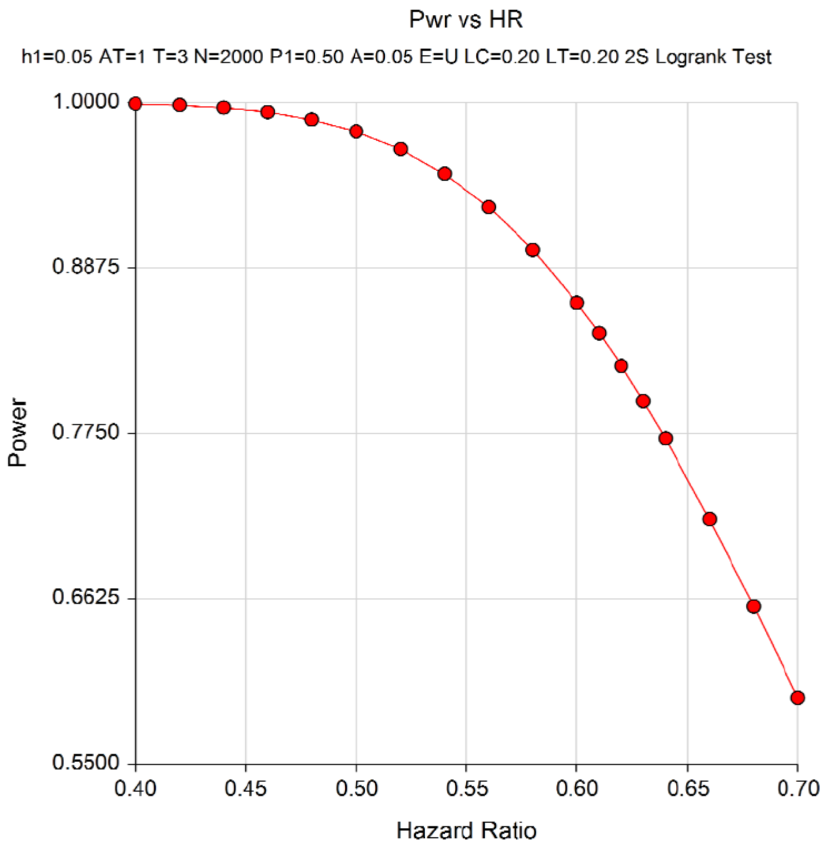

CONF

Appendix 5. **Questionnaires**

EQ-5D-3L

By placing a tick in one box in each group below, please indicate which statements best describe your own health state today.

Mobility

I have no problems in walking about	❑
I have some problems in walking about	❑
I am confined to bed	❑

Self-Care

I have no problems with self-care	❑
I have some problems washing or dressing myself	❑
I am unable to wash or dress myself	❑

Usual Activities (*e.g. work, study, housework, family or leisure activities)*

I have no problems with performing my usual activities	❑
I have some problems with performing my usual activities	❑
I am unable to perform my usual activities	❑

Pain/Discomfort

I have no pain or discomfort	❑
I have moderate pain or discomfort	❑
I have extreme pain or discomfort	❑

Anxiety/Depression

I am not anxious or depressed	❑
I am moderately anxious or depressed	❑
I am extremely anxious or depressed	❑

CONFIDENTIAL

Hong Kong (English) © 2005 EuroQol Group. EQ-5D™ is a trade mark of the EuroQol Group

Best imaginable health state

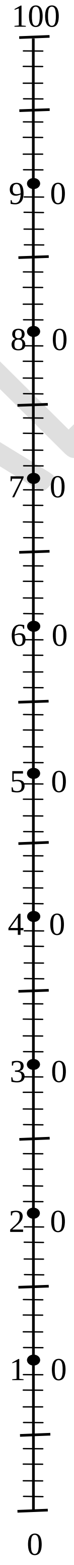

Worst imaginable health state

To help people say how good or bad a health state is, we have drawn a scale (rather like a thermometer) on which the best state you can imagine is marked 100 and the worst state you can imagine is marked 0.

We would like you to indicate on this scale how good or bad your own health is today, in your opinion. Please do this by drawing a line from the black box on the left side of the scale to whichever point on the scale that indicates how good or bad your health state is today.

Your own health state today

CONFIDENTIAL

Hong Kong (English) © 2005 EuroQol Group. EQ-5D™ is a trade mark of the EuroQol Group

WHOQOL – BREF

Please read each question, assess your feelings, and circle the number on the scale for each question that gives the best answer for you.

		Very poor	Poor	Neither poor nor good	Good	Very good
1	How would you rate your quality of life?	1	2	3	4	5

		Very dissatisfied	Dissatisfied	Neither satisfied nor dissatisfied	Satisfied	Very satisfied
2	How satisfied are you with your health?	1	2	3	4	5

The following questions ask about **how much** you have experienced certain things in the last two weeks.

		Not at all	A little	A moderate amount	Very much	An extreme amount
3	To what extent do you feel that physical pain prevents you from doing what you need to do?	1	2	3	4	5
4	How much do you need any medical treatment to function in your daily life?	1	2	3	4	5
5	How much do you enjoy life?	1	2	3	4	5
6	To what extent do you feel your life to be meaningful?	1	2	3	4	5

		Not at all	A little	A moderate amount	Very much	An extreme amount
7	How well are you able to concentrate?	1	2	3	4	5
8	How safe do you feel in your daily life?	1	2	3	4	5
9	How healthy is your physical environment?	1	2	3	4	5

The following questions ask about **how completely** your experience or were able to do certain things in the last two weeks.

		Not at all	A little	Moderately	Mostly	Completely
10	Do you have enough energy for everyday life?	1	2	3	4	5
11	Are you able to accept your bodily appearance?	1	2	3	4	5
12	Have you enough money to meet your needs?	1	2	3	4	5

CONFIDENTIAL

13	How available to you is the information that you need in your day-to-day life?	1	2	3	4	5
14	To what extent do you have the opportunity for leisure activities?	1	2	3	4	5

		Very poor	Poor	Neither poor nor good	Good	Very good
15	How well are you able to get around?	1	2	3	4	5

The following questions ask you to say how good or satisfied you have felt about various aspects of your life over the last two weeks.

		Very dissatisfied	Dissatisfied	Neither satisfied nor dissatisfied	Satisfied	Very satisfied
16	How satisfied are you with your sleep?	1	2	3	4	5
17	How satisfied are you with your ability to perform your daily living activities?	1	2	3	4	5
18	How satisfied are you with your capacity for work?	1	2	3	4	5
19	How satisfied are you with yourself?	1	2	3	4	5
20	How satisfied are you with your personal relationships?	1	2	3	4	5
21	How satisfied are you with your sex life?	1	2	3	4	5
22	How satisfied are you with the support you get from your friends?	1	2	3	4	5
23	How satisfied are you with the conditions of your living place?	1	2	3	4	5
24	How satisfied are you with your access to health services?	1	2	3	4	5
25	How satisfied are you with your transport?	1	2	3	4	5

The following question refers to **how often** you have felt or experienced certain things in the last two weeks.

		Never	Seldom	Quite often	Very often	Always
26	How often do you have negative feelings such as blue mood, despair, anxiety, depression?	1	2	3	4	5

CONFIDENTIAL

CONFIDENTIAL

<u>Time Trade Off Question</u>

Imagine that you are told that you have 10 years left to live. In connection with this you are also told that you can choose to live these 10 years in your current health state or that you can choose to give up some life years to live for a shorter period in full health. Indicate with a cross on the line the number of years in full health that you think is of equal value to 10 years in your current health state.

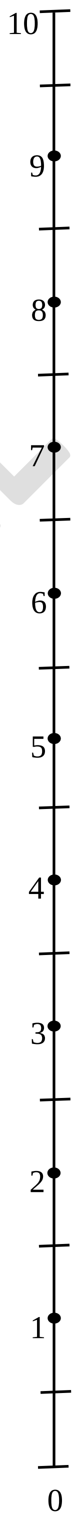

References

1. Burstrom K, Johannesson M. and Diderichsen F., 2006, A comparison of individual and social time trade-off values for health states in the general population, Health Policy 76(3), 359-370.

2. Torrance G., 1986, Measurement of health state utilities for economic appraisal, Journal of Health Economics 5(1), 1-30.

PATIENT HEALTH QUESTIONNAIRE - 9 (PHQ - 9)

**Over the last 2 weeks, how often have you been bothered by any of the following problems?** *(Use "✓" to indicate your answer)*	**Not at all**	**Several days**	**More than half the days**	**Nearly every day**
**1.** Little interest or pleasure in doing things	0	1	2	3
**2.** Feeling down, depressed, or hopeless	0	1	2	3
**3.** Trouble falling or staying asleep, or sleeping too much	0	1	2	3
**4.** Feeling tired or having little energy	0	1	2	3
**5.** Poor appetite or overeating	0	1	2	3
**6.** Feeling bad about yourself — or that you are a failure or have let yourself or your family down	0	1	2	3
**7.** Trouble concentrating on things, such as reading the newspaper or watching television	0	1	2	3
**8.** Moving or speaking so slowly that other people could have noticed? Or the opposite — being so fidgety or restless that you have been moving around a lot more than usual	0	1	2	3
**9.** Thoughts that you would be better off dead or of hurting yourself in some way	0	1	2	3

FOR OFFICE CODING__0 + ______ + ______ + ______

=Total Score: ______

CONFIDENTIAL

Developed by Drs. Robert L. Spitzer, Janet B.W. Williams, Kurt Kroenke and colleagues, with an educational grant from Pfizer Inc. No permission required to reproduce, translate, display or distribute.

DASS21

Please read each statement and circle a number 0, 1, 2 or 3 which indicates how much the statement applied to you *over the past week*. There are no right or wrong answers. Do not spend too much time on any statement.

The rating scale is as follows:

0 Did not apply to me at all
1 Applied to me to some degree, or some of the time
2 Applied to me to a considerable degree, or a good part of time
3 Applied to me very much, or most of the time

1.	I found it hard to wind down	0	1	2	3
2.	I was aware of dryness of my mouth	0	1	2	3
3.	I couldn't seem to experience any positive feeling at all	0	1	2	3
4.	I experienced breathing difficulty (eg, excessively rapid breathing, breathlessness in the absence of physical exertion)	0	1	2	3
5.	I found it difficult to work up the initiative to do things	0	1	2	3
6.	I tended to over-react to situations	0	1	2	3
7.	I experienced trembling (eg, in the hands)	0	1	2	3
8.	I felt that I was using a lot of nervous energy	0	1	2	3
9.	I was worried about situations in which I might panic and make a fool of myself	0	1	2	3
10.	I felt that I had nothing to look forward to	0	1	2	3
11.	I found myself getting agitated	0	1	2	3
12.	I found it difficult to relax	0	1	2	3
13.	I felt down-hearted and blue	0	1	2	3
14.	I was intolerant of anything that kept me from getting on with what I was doing	0	1	2	3
15.	I felt I was close to panic	0	1	2	3
16.	I was unable to become enthusiastic about anything	0	1	2	3
17.	I felt I wasn't worth much as a person	0	1	2	3
18.	I felt that I was rather touchy	0	1	2	3
19.	I was aware of the action of my heart in the absence of physical exertion (eg, sense of heart rate increase, heart missing a beat)	0	1	2	3
20.	I felt scared without any good reason	0	1	2	3
21.	I felt that life was meaningless	0	1	2	3

CONFIDENTIAL
