# Supplementary material for: Effect of a Web-Based Management Guide on Risk Factors in Patients With Type 2 Diabetes and Diabetic Kidney Disease: A JADE Randomized Clinical Trial
Source: JAMA Netw Open. 2022 Mar 25;5(3):e223862. doi: 10.1001/jamanetworkopen.2022.3862 (PMC8956973; doi:10.1001/jamanetworkopen.2022.3862)
Supplement: Supplement 2. — eTable 1. Distribution of Randomized Patients by Study Sites and Regions eTable 2. Definition of Clinical Events eTable 3. Comparison of Baseline Clinical Characteristics Between Patients Who Did or Did Not Return for End-of-Study Assessment eTable 4. Comparison of Baseline Clinical Characteristics by Target Attainment at 12 Months in the Intention-to-Treat Population eTable 5. Number of Patients With Incident Clinical Events by Group Randomization in the Intention-to-Treat Population eTable 6. Number of Patients With Incident Non-Fatal Clinical Events by Target Attainment at 12 Months in the Intention-to-Treat Population eTable 7. Poisson Regression to Show the Association Between Incident Non-Fatal Clinical Events and Target Attainment at 12 Months in the Intention-to-Treat Population eTable 8. Poisson Regression to Show the Association Between Incident Non-Fatal Clinical Events and Target Attainment at 12 Months in the Per-Protocol Population eTable 9. Baseline Clinical Characteristics of Patients Who Adhered to the Study Protocol and Returned for End-of-Study Assessment by Group Randomization in the Per-Protocol Population eTable 10. Poisson Regression to Show the Association of Assignment to Team-Based Empowered Care Compared With Empowered Care and Usual Care on Attainment to Multiple Treatment Targets at 12 Months in the Per-Protocol Population eTable 11. Number of Patients With Incident Clinical Events by Group Randomization at 12 Months in the Per-Protocol Population eTable 12. Number of Patients With Incident Non-Fatal Clinical Events by Target Attainment at 12 Months in the Per-Protocol Population eFigure. Changes in the Proportion of Patients Attaining at Least 3 Treatment Targets at 12 Months in the Per-Protocol Population [file jamanetwopen-e223862-s002.pdf]

Supplementary Online Content

Chan JCN, Thewjitcharoen Y, Nguyen TK, et al. Effect of a web-based management guide on risk factors in patients with type 2 diabetes and diabetic kidney disease: a JADE randomized clinical trial. *JAMA Netw Open*. 2022;5(3):e223862. doi:10.1001/jamanetworkopen.2022.3862

eTable 1. Distribution of Randomized Patients by Study Sites and Regions
eTable 2. Definition of Clinical Events
eTable 3. Comparison of Baseline Clinical Characteristics Between Patients Who Did or Did Not Return for End-of-Study Assessment
eTable 4. Comparison of Baseline Clinical Characteristics by Target Attainment at 12 Months in the Intention-to-Treat Population
eTable 5. Number of Patients With Incident Clinical Events by Group Randomization in the Intention-to-Treat Population
eTable 6. Number of Patients With Incident Non-Fatal Clinical Events by Target Attainment at 12 Months in the Intention-to-Treat Population
eTable 7. Poisson Regression to Show the Association Between Incident Non-Fatal Clinical Events and Target Attainment at 12 Months in the Intention-to-Treat Population
eTable 8. Poisson Regression to Show the Association Between Incident Non-Fatal Clinical Events and Target Attainment at 12 Months in the Per-Protocol Population
eTable 9. Baseline Clinical Characteristics of Patients Who Adhered to the Study Protocol and Returned for End-of-Study Assessment by Group Randomization in the Per-Protocol Population
eTable 10. Poisson Regression to Show the Association of Assignment to Team-Based Empowered Care Compared With Empowered Care and Usual Care on Attainment to Multiple Treatment Targets at 12 Months in the Per-Protocol Population
eTable 11. Number of Patients With Incident Clinical Events by Group Randomization at 12 Months in the Per-Protocol Population
eTable 12. Number of Patients With Incident Non-Fatal Clinical Events by Target Attainment at 12 Months in the Per-Protocol Population
eFigure. Changes in the Proportion of Patients Attaining at Least 3 Treatment Targets at 12 Months in the Per-Protocol Population

This supplementary material has been provided by the authors to give readers additional information about their work.

© 2022 Chan JCN et al. *JAMA Network Open.*

eTable 1. Distribution of randomized patients by study sites and regions

Region	Site*	Number of patients randomized
China	The Fourth Affiliated Hospital of China Medical University	113
Hong Kong	Alice Ho Miu Ling Nethersole Hospital	100
Hong Kong	Prince of Wales Hospital	403
Malaysia	Universiti Sains Malaysia	89
Malaysia	Department of Primary Care Medicine, University of Malaya Medical Centre	297
Malaysia	Department of Medicine, University of Malaya Medical Centre	300
South Korea	Seoul St. Mary’s Hospital	106
South Korea	Hallym University Dongtan Sacred Heart Hospital	249
Taiwan	Taipei Veterans General Hospital	250
Thailand	Theptarin Hospital	162
Vietnam	Medic Medical Center, Ho Chi Minh, Vietnam	324

*2 sites did not enroll any patients due to administrative delay.

© 2022 Chan JCN et al. *JAMA Network Open.*

eTable 2. Definition of clinical events

**Cardiovascular disease**	a) coronary artery disease<br>• acute coronary syndrome<br>• percutaneous transluminal coronary angioplasty<br>• coronary artery bypass graft<br><br>b) cerebrovascular disease<br>• ischemic stroke<br>• hemorrhagic stroke<br>• percutaneous transluminal cerebral angioplasty<br>• carotid endarterectomy<br>• carotid artery stenting<br><br>c) peripheral artery disease<br>• peripheral percutaneous transluminal angioplasty to lower-extremity arteries<br>• non-traumatic lower-extremity amputation<br>• ankle-brachial index<0.9
**End-stage kidney disease**	a) dialysis<br>b) kidney transplant, or<br>c) eGFR <15 mL/min/1.73m^2
**Cancer**	Cancer arising from any organs and at all stages

© 2022 Chan JCN et al. *JAMA Network Open.*

eTable 3. Comparison of baseline clinical characteristics between patients who did or did not return for end-of-study assessment

Variable	Patients who did return (n=2067)	Patients who did not return (n=326)	p-value
Number by group randomization			
Usual care	691	104	
Empowered care	697	105	
Team-based empowered care	679	117	
Sociodemographic characteristics			
Age, mean (SD), y	67.7 (9.9)	67.6 (9.6)	0.85
Men, n (%)	1085 (52.5%)	182 (55.8%)	0.29
Women, n (%)	982 (47.5%)	144 (44.2%)	0.29
Race and ethnicity, n (%)			<0.001
Chinese	980 (47.4%)	88 (27.0%)	
Indian	170 (8.2%)	20 (6.2%)	
Korean	306 (14.8%)	49 (15.0%)	
Malay	198 (9.6%)	97 (29.8%)	
Thai	137 (6.6%)	24 (7.4%)	
Vietnamese	268 (13.0%)	48 (14.7%)	
Other	5 (0.3%)	0 (0%)	
≥College-level education, n (%)	414 (20.0%)	70 (21.5%)	0.62
Smoking status, n (%)			<0.001
Current	186 (9.0%)	30 (9.2%)	
Previous	357 (17.3%)	89 (27.3%)	
Diabetes and metabolic profile, mean (SD)			
Diabetes duration, y	16.5 (9.9)	16.0 (9.7)	0.42
Age at diagnosis, y	51.2 (11.7)	51.5 (11.6)	0.61
BMI	26.9 (4.7)	26.7 (5.3)	0.91
Waist circumference, cm			
Men	96.0 (11.2)	94.9 (10.5)	0.19
Women	92.9 (11.6)	92.3 (12.8)	0.64
BP, mmHg			
Systolic	138.0 (18.2)	139.0 (20.4)	0.63
Diastolic	74.3 (11.1)	74.4 (11.2)	0.85
HbA_{1c} level, %	7.8 (1.6)	8.3 (1.9)	<0.001
HbA_{1c} level, mmol/mol	61.7 (17.1)	67.5 (21.1)	<0.001
Fasting plasma glucose level, mmol/L	8.1 (3.3)	8.7 (3.9)	0.01
Total cholesterol level, mmol/L	4.3 (1.1)	4.5 (1.2)	0.05
Triglyceride level, mmol/L	1.9 (1.3)	1.9 (1.2)	0.33
HDL-cholesterol level, mmol/L	1.2 (0.4)	1.2 (0.4)	0.01
LDL-cholesterol level, mmol/L	2.3 (1.1)	2.5 (1.0)	0.02
eGFR, mL/min/1.73m^2	49.9 (16.5)	48.8 (15.3)	0.21
Urinary ACR, mg/mmol	57.8 (136.0)	93.5 (189.0)	0.001
General obesity, n (%)	1,265 (61.2%)	202 (61.8%)	0.86
Hypertension, n (%)	1,942 (94.0%)	307 (94.2%)	0.99
Dyslipidemia, n (%)	1,977 (95.6%)	311 (95.3%)	0.89
Complications at baseline, n (%)			
eGFR <65 mL/min/1.73m^2	1,850 (89.5%)	294 (90.1%)	0.81
Macroalbuminuria	716 (34.6%)	150 (46.2%)	0.004
Coronary artery disease	434 (21.0%)	72 (22.1%)	0.71
Stroke	166 (8.0%)	34 (10.4%)	0.18
Peripheral artery disease	113 (5.5%)	48 (14.7%)	<0.001

© 2022 Chan JCN et al. *JAMA Network Open.*

Variable	Patients who did return (n=2067)	Patients who did not return (n=326)	p-value
Any cardiovascular disease	609 (29.5%)	129 (39.6%)	<0.001
Congestive heart failure	77 (3.7%)	18 (5.5%)	0.16
Cancer	104 (5.0%)	15 (4.6%)	0.85
Diabetic retinopathy	411 (19.9%)	58 (17.7%)	0.39
Peripheral neuropathy	509 (24.6%)	89 (27.3%)	0.33
Medication use at baseline, n (%)			
RAAS inhibitors	1,439 (69.6%)	226 (69.3%)	0.97
BP-lowering drugs	1,732 (83.8%)	271 (83.1%)	0.83
Lipid-lowering drugs	1,600 (77.4%)	251 (77.0%)	0.93
Non-insulin glucose-lowering drugs	1,719 (83.2%)	258 (79.1%)	0.09
Insulin	968 (46.8%)	167 (51.2%)	0.16
Diabetes self-care in past 3 months, n (%)			
SMBG at least once weekly	1,184 (57.3%)	195 (59.8%)	0.45
Physical exercise at least 3 times per week	835 (40.4%)	116 (35.7%)	0.13
Adherence to balanced diet	1,671 (80.8%)	257 (78.9%)	0.46
At least 2 self-care activities	1,348 (65.2%)	200 (61.3%)	0.21
Metabolic targets, n (%)			
HbA_{1c} level <7.0% (53 mmol/mol)	694 (33.6%)	89 (27.2%)	0.03
BP <130/80 mmHg	583 (28.2%)	89 (27.2%)	0.77
LDL-cholesterol level <1.8 mmol/L	558 (27.0%)	78 (23.9%)	0.28
Triglyceride level <1.7 mmol/L	1,104 (53.4%)	185 (56.8%)	0.29
At least 3 treatment targets	730 (35.3%)	101 (30.9%)	0.14

Footnotes: Results are presented as mean (standard deviation) or number (percentage). General obesity was defined as BMI ≥25 kg/m^2. Hypertension was defined as blood pressure ≥130/80 mmHg and/or usage of blood pressure-lowering drugs. Dyslipidemia was defined as LDL-cholesterol ≥1.8 mmol/L and/or usage of lipid-lowering drugs. ACR, albumin-to-creatinine ratio; BMI, body mass index (calculated as weight in kilograms divided by height in meter squared); BP, blood pressure; eGFR, estimated glomerular filtration rate; HbA_{1c}, glycated hemoglobin; HDL, high-density lipoprotein; LDL, low-density lipoprotein; RAAS, renin-angiotensin-aldosterone system; SMBG, self-monitoring of blood glucose.

© 2022 Chan JCN et al. *JAMA Network Open.*

eTable 4. Comparison of baseline clinical characteristics by target attainment at 12 months in the intention-to-treat population

Variable	Patients who attained <3 targets (n=1448)	Patients who attained ≥3 targets (n=945)	p-value
Number by group assignment			
Usual care	491	304	
Empowered care	516	286	
Team-based empowered care	441	355	
Sociodemographic characteristics			
Age, mean (SD), y	66.7 (10.4)	69.1 (9.7)	<0.001
Men, n (%)	720 (49.7%)	547 (57.9%)	<0.001
Women, n (%)	728 (50.3%)	398 (42.1%)	<0.001
Race and ethnicity, n (%)			<0.001
Chinese	641 (44.3%)	428 (45.3%)	
Indian	118 (8.2%)	72 (7.6%)	
Korean	181 (12.5%)	174 (18.4%)	
Malay	210 (14.5%)	85 (9%)	
Thai	70 (4.8%)	91 (9.6%)	
Vietnamese	225 (15.5%)	91 (9.6%)	
Other	2 (0.2%)	3 (0.3%)	
≥College-level education, n (%)	276 (19.1%)	208 (22%)	0.12
Smoking status, n (%)			0.03
Current	144 (9.9%)	73 (7.7%)	
Previous	248 (17.1%)	200 (21.2%)	
Diabetes and metabolic profile, mean (SD)			
Diabetes duration, y	16.3 (10.2)	16.6 (10.5)	0.47
Age at diagnosis, y	50.4 (11.8)	52.5 (11.9)	<0.001
BMI	27.2 (5.1)	26.4 (4.5)	<0.001
Waist circumference, cm			
Men	95.9 (11.5)	95.7 (11.1)	0.81
Women	93.2 (12.4)	92.1 (11.8)	0.16
BP, mmHg			
Systolic	140.3 (19.6)	135.9 (19.3)	<0.001
Diastolic	75.3 (11.7)	72.7 (10.8)	<0.001
HbA_{1c} level, %	8.2 (1.7)	7.4 (1.4)	<0.001
HbA_{1c} level, mmol/mol	66.1 (19.0)	57.0 (15.8)	<0.001
Fasting plasma glucose level, mmol/L	8.5 (3.7)	7.6 (2.9)	<0.001
Total cholesterol level, mmol/L	4.5 (1.2)	4.1 (1.0)	<0.001
Triglyceride level, mmol/L	2.2 (1.5)	1.6 (1.0)	<0.001
HDL-cholesterol level, mmol/L	1.2 (0.4)	1.2 (0.4)	<0.001
LDL-cholesterol level, mmol/L	2.5 (1.1)	2.2 (1.2)	<0.001
eGFR, mL/min/1.73m^2	49.7 (17.1)	49.8 (16.1)	0.86
Urinary ACR, mg/mmol	71.7 (154.6)	48.1 (127.4)	<0.001
General obesity, n (%)	919 (63.5%)	547 (57.9%)	0.01
Hypertension, n (%)	1363 (94.1%)	887 (93.9%)	0.77
Dyslipidemia, n (%)	1393 (96.2%)	895 (94.7%)	0.14

© 2022 Chan JCN et al. *JAMA Network Open.*

Variable	Patients who attained <3 targets (n=1448)	Patients who attained ≥3 targets (n=945)	p-value
Complications at baseline, n (%)			
eGFR <65 mL/min/1.73m^2	1285 (88.7%)	859 (90.9%)	0.13
Macroalbuminuria	573 (39.5%)	290 (30.6%)	<0.001
Coronary artery disease	306 (21.1%)	200 (21.1%)	0.73
Stroke	121 (8.4%)	79 (8.3%)	0.86
Peripheral artery disease	92 (6.3%)	73 (7.7%)	0.33
Any cardiovascular disease	449 (31.0%)	290 (30.7%)	0.71
Congestive heart failure	57 (4.0%)	38 (4%)	0.83
Cancer	62 (4.3%)	57 (6%)	0.11
Diabetic retinopathy	294 (20.3%)	175 (18.5%)	0.36
Peripheral neuropathy	390 (27%)	208 (22%)	0.01
Medication use at baseline, n (%)			
RAAS inhibitors	940 (64.9%)	725 (76.7%)	<0.001
BP-lowering drugs	1174 (81.1%)	829 (87.7%)	<0.001
Lipid-lowering drugs	1109 (76.6%)	742 (78.5%)	0.36
Non-insulin glucose-lowering drugs	1177 (81.3%)	800 (84.7%)	0.05
Insulin	751 (51.9%)	384 (40.6%)	<0.001
Diabetes self-care in past 3 months, n (%)			
SMBG at least once weekly	850 (58.7%)	527 (55.7%)	0.20
Physical exercise at least 3 times per week	540 (37.3%)	414 (43.8%)	0.003
Adherence to balanced diet	1145 (79.1%)	782 (82.8%)	0.05
At least 2 self-care activities	909 (62.8%)	638 (67.5%)	0.04
Metabolic targets, n (%)			
HbA_{1c} level <7.0% (53 mmol/mol)	342 (23.6%)	441 (46.6%)	<0.001
BP <130/80 mmHg	334 (23.1%)	336 (35.6%)	<0.001
LDL-cholesterol level <1.8 mmol/L	313 (21.6%)	324 (34.3%)	<0.001
Triglyceride level <1.7 mmol/L	626 (43.2%)	664 (70.3%)	<0.001

Footnotes: Results are presented as mean (standard deviation) or number (percentage). General obesity was defined as BMI ≥25 kg/m^2. Hypertension was defined as blood pressure ≥130/80 mmHg and/or usage of blood pressure-lowering drugs. Dyslipidemia was defined as LDL-cholesterol ≥1.8 mmol/L and/or usage of lipid-lowering drugs. ACR, albumin-to-creatinine ratio; BMI, body mass index (calculated as weight in kilograms divided by height in meter squared); BP, blood pressure; eGFR, estimated glomerular filtration rate; HbA_{1c}, glycated hemoglobin; HDL, high-density lipoprotein; LDL, low-density lipoprotein; RAAS, renin-angiotensin-aldosterone system; SMBG, self-monitoring of blood glucose.

© 2022 Chan JCN et al. *JAMA Network Open.*

eTable 5. Number of patients with incident clinical events by group randomization in the intention-to-treat population

	Total	Usual care	Empowered care	Team-based empowered care	p-value
**Any events**					
Total	1526	514	517	495	
n (%)	187 (12.2%)	56 (10.9%)	66 (12.7%)	65 (13.2%)	0.52
**Cardiovascular disease**					
Total	1657	555	557	545	
n (%)	98 (5.9%)	30 (5.4%)	36 (6.5%)	32 (5.9%)	0.76
**Congestive heart failure**					
Total	2298	768	763	767	
n (%)	10 (0.4%)	4 (0.5%)	1 (0.1%)	5 (0.7%)	0.27
**End-stage kidney disease**					
Total	2387	795	799	793	
n (%)	70 (2.9%)	15 (1.9%)	26 (3.2%)	29 (3.6%)	0.21
**All-site cancer**					
Total	2274	760	760	754	
n (%)	9 (0.4%)	4 (0.5%)	3 (0.4%)	2 (0.3%)	0.72
**All-cause death**					
Total	2393	795	802	796	
n (%)	33 (1.4%)	12 (1.5%)	13 (1.6%)	8 (1.0%)	0.53

Footnotes: n (%), number of patients (percentage). Any events are defined as the occurrence of cardiovascular disease, congestive heart failure, end-stage kidney disease, all-site cancer and/or all-cause death. Cardiovascular disease is defined as the occurrence of coronary artery disease, stroke and/or peripheral artery disease. End-stage kidney disease is defined as estimated glomerular filtration rate <15 $mL/min/1.73m^2$ or a need for kidney replacement therapy.

© 2022 Chan JCN et al. *JAMA Network Open.*

eTable 6. Number of patients with incident non-fatal clinical events by target attainment at 12 months in the intention-to-treat population

	Total	Attained <3 treatment targets	Attained ≥3 treatment targets	p-value
**Any events**				
Total	1526	923	603	
n (%)	185 (13.8%)	134 (14.5%)	51 (8.4%)	0.004
**Cardiovascular disease**				
Total	1657	1001	656	
n (%)	98 (6.3%)	74 (7.4%)	24 (3.7%)	0.004
**Congestive heart failure**				
Total	2298	1391	908	
n (%)	10 (0.4%)	5 (0.4%)	5 (0.6%)	0.72
**End-stage kidney disease**				
Total	2387	1444	943	
n (%)	72 (3.1%)	51 (3.5%)	22 (2.3%)	0.31
**All-site cancer**				
Total	2274	1386	888	
n (%)	9 (0.4%)	6 (0.4%)	3 (0.3%)	0.99

Footnotes: n (%), number of patients (percentage). Any events are defined as the occurrence of non-fatal cardiovascular disease, congestive heart failure, end-stage kidney disease, and/or all-site cancer. Cardiovascular disease is defined as the occurrence of coronary artery disease, stroke and/or peripheral artery disease. End-stage kidney disease is defined as estimated glomerular filtration rate <15 mL/min/1.73m^2 or a need for kidney replacement therapy.

© 2022 Chan JCN et al. *JAMA Network Open.*

eTable 7. Poisson regression to show the association between incident non-fatal clinical events and target attainment at 12 months in the intention-to-treat population

	Attainment of ≥3 treatment targets vs <3 treatment targets at 12 months			
	**Model 1**	**p-value**	**Model 2**	**p-value**
Any events	0.80 (0.55-1.18)	0.27	0.79 (0.54-1.17)	0.24
Cardiovascular disease	0.65 (0.39-1.09)	0.10	0.64 (0.38-1.08)	0.10
Congestive heart failure	1.64 (0.43-6.20)	0.47	1.63 (0.42-6.28)	0.48
End-stage kidney disease	0.77 (0.40-1.50)	0.45	0.78 (0.40-1.54)	0.48
All-site cancer	0.57 (0.13-2.50)	0.45	0.57 (0.13-2.53)	0.46

Footnotes: Results are presented as adjusted risk ratio with 95% confidence interval, with patients who attained <3 treatment targets as the referent. Any events are defined as the occurrence of cardiovascular disease, congestive heart failure, end-stage kidney disease, all-site cancer and/or all-cause death. Cardiovascular disease is defined as the occurrence of coronary artery disease, stroke and/or peripheral artery disease. End-stage kidney disease is defined as estimated glomerular filtration rate <15 mL/min/1.73m^2 or a need for kidney replacement therapy.

Model 1: adjusted for age, sex, diabetes duration, site and target attainment at baseline.

Model 2: Model 1 plus self-care (self-monitoring of blood glucose at least weekly, physical exercise at least 3 times per week and adherence to balanced diet in past 3 months) at baseline

© 2022 Chan JCN et al. *JAMA Network Open.*

eTable 8. Poisson regression to show the association between incident non-fatal clinical events and target attainment at 12 months in the per-protocol population

	Attainment of ≥3 treatment targets vs <3 treatment targets at 12 months			
	**Model 1**	**p-value**	**Model 2**	**p-value**
Any events	0.84 (0.52-1.34)	0.45	0.84 (0.51-1.38)	0.49
Cardiovascular disease	0.68 (0.39-1.18)	0.17	0.75 (0.43-1.33)	0.33
Congestive heart failure	1.70 (0.44-6.50)	0.44	4.27 (0.40-45.70)	0.23
End-stage kidney disease	0.82 (0.37-1.83)	0.63	0.82 (0.35-1.93)	0.66
All-site cancer	0.77 (0.16-3.74)	0.75	0.98 (0.18-5.39)	0.98

Footnotes: Results are presented as adjusted risk ratio with 95% confidence interval, with patients who attained <3 treatment targets as the referent. Any events are defined as the occurrence of cardiovascular disease, congestive heart failure, end-stage kidney disease and/or all-site cancer. Cardiovascular disease is defined as the occurrence of coronary artery disease, stroke and/or peripheral artery disease. End-stage kidney disease is defined as estimated glomerular filtration rate <15 mL/min/1.73m^2 or a need for kidney replacement therapy.

Model 1: adjusted for age, sex, diabetes duration, site and target attainment at baseline.

Model 2: Model 1 plus self-care (self-monitoring of blood glucose at least weekly, physical exercise at least 3 times per week and adherence to balanced diet in past 3 months) at baseline

© 2022 Chan JCN et al. *JAMA Network Open.*

eTable 9. Baseline clinical characteristics of patients who adhered to the study protocol and returned for end-of-study assessment by group randomization in the per-protocol population

Variable	Total No. of patients (n=1695)	Usual care group (n=625)	Empowered care group (n=559)	Team-based empowered care group (n=511)	p-value
Sociodemographic characteristics					
Age, mean (SD), y	68.4 (9.5)	68.6 (9.7)	68.4 (9.8)	68.1 (8.9)	0.78
Men, n (%)	886 (52.3%)	323 (51.7%)	285 (51.0%)	278 (54.4%)	0.50
Women, n (%)	809 (47.7%)	302 (48.3%)	274 (49.0%)	233 (45.6%)	0.50
Race and ethnicity, n (%)					<0.001
Chinese	750 (45.7%)	291 (48%)	239 (44.2%)	220 (44.5%)	
Indian	144 (8.8%)	45 (7.4%)	51 (9.4%)	48 (9.7%)	
Korean	271 (16.5%)	91 (15%)	89 (16.5%)	91 (18.4%)	
Malay	156 (9.5%)	56 (9.2%)	49 (9.1%)	51 (10.3%)	
Thai	85 (5.2%)	44 (7.3%)	35 (6.5%)	6 (1.2%)	
Vietnamese	230 (14.0%)	77 (12.7%)	75 (13.9%)	78 (15.8%)	
Other	5 (0.3%)	2 (0.2%)	3 (0.6%)	0	
≥College-level education, n (%)	311 (18.9%)	111 (18.4%)	97 (17.8%)	103 (20.7%)	0.46
Smoking status, n (%)					0.86
Current	150 (8.9%)	59 (9.5%)	48 (8.6%)	43 (8.4%)	
Previous	336 (19.9%)	117 (18.8%)	111 (20%)	108 (21.2%)	
Diabetes and metabolic profile, mean (SD)					
Diabetes duration, y	16.9 (9.7)	16.5 (9.7)	17.1 (9.4)	17.2 (9.9)	0.44
Age at diagnosis, y	51.6 (11.3)	52.2 (11.3)	51.5 (11.7)	50.9 (10.9)	0.17
BMI	26.8 (4.6)	27 (4.4)	26.9 (4.9)	26.4 (4.5)	0.10
Waist circumference, cm					
Men	95.9 (10.9)	96.3 (10.4)	96 (11.8)	95.2 (10.7)	0.50
Women	92.8 (11.2)	93.5 (10.8)	92.8 (11.4)	91.9 (11.5)	0.28
BP, mmHg					
Systolic	138.4 (18)	138.3 (18.9)	138.8 (16.5)	138.2 (18.5)	0.84
Diastolic	73.8 (10.9)	73.7 (10.8)	74.1 (10.5)	73.6 (11.6)	0.70
HbA_{1c} level, %	7.8 (1.5)	7.7 (1.5)	7.8 (1.5)	7.8 (1.5)	0.68
HbA_{1c} level, mmol/mol	62.0 (16.4)	61.0 (16.4)	62.0 (16.4)	62.0 (16.4)	0.68
Fasting plasma glucose level, mmol/L	8.1 (3.2)	8.2 (3.1)	8 (3)	8.1 (3.4)	0.63
Total cholesterol level, mmol/L	4.4 (0.9)	4.4 (1)	4.4 (0.9)	4.4 (1)	0.99
Triglyceride level, mmol/L	1.9 (1.4)	1.9 (1.3)	2 (1.5)	1.9 (1.3)	0.68
HDL-cholesterol level, mmol/L	1.2 (0.4)	1.2 (0.4)	1.2 (0.4)	1.2 (0.5)	0.19

© 2022 Chan JCN et al. *JAMA Network Open.*

Variable	Total No. of patients (n=1695)	Usual care group (n=625)	Empowered care group (n=559)	Team-based empowered care group (n=511)	p-value
LDL-cholesterol level, mmol/L	2.3 (1.1)	2.3 (0.8)	2.4 (1.2)	2.3 (1.3)	0.44
eGFR, mL/min/1.73m^2	48 (13.1)	48.4 (13.2)	47.6 (12.9)	47.8 (13.1)	0.60
Urinary ACR, mg/mmol	62.3 (124.2)	58.2 (102.9)	59.8 (120)	70.1 (150.2)	0.31
General obesity, n (%)	1006 (59.9%)	403 (65.1%)	327 (59.1%)	276 (54.3%)	0.001
Hypertension, n (%)	1595 (94.3%)	586 (93.9%)	530 (95.0%)	479 (93.9%)	0.68
Dyslipidemia, n (%)	1579 (96.0%)	583 (96.2%)	529 (97.2%)	467 (94.3%)	0.06
Complications at baseline, n (%)					
eGFR <65 mL/min/1.73m^2	1622 (95.7%)	594 (95%)	537 (96.1%)	491 (96.1%)	0.60
Macroalbuminuria	492 (36.3%)	188 (37.7%)	164 (36.2%)	140 (34.7%)	0.64
Coronary artery disease	363 (21.4%)	129 (20.6%)	118 (21.1%)	116 (22.7%)	0.69
Stroke	129 (7.6%)	49 (7.8%)	44 (7.9%)	36 (7%)	0.85
Peripheral artery disease	90 (5.3%)	35 (5.6%)	32 (5.7%)	23 (4.5%)	0.62
Any cardiovascular disease	504 (29.7%)	183 (29.3%)	166 (29.7%)	155 (30.3%)	0.93
Congestive heart failure	62 (3.7%)	20 (3.2%)	24 (4.3%)	18 (3.5%)	0.60
Cancer	83 (4.9%)	24 (3.8%)	29 (5.2%)	30 (5.9%)	0.27
Diabetic retinopathy	331 (19.6%)	127 (20.4%)	98 (17.7%)	106 (20.8%)	0.36
Peripheral neuropathy	411 (24.2%)	141 (22.6%)	152 (27.2%)	118 (23.1%)	0.14
Medication use at baseline, n (%)					
RAAS inhibitors	1201 (70.9%)	442 (70.7%)	396 (70.8%)	363 (71%)	0.99
BP-lowering drugs	1439 (84.9%)	535 (85.6%)	467 (83.5%)	437 (85.5%)	0.55
Lipid-lowering drugs	1325 (78.2%)	496 (79.4%)	437 (78.2%)	392 (76.7%)	0.56
Non-insulin glucose-lowering drugs	1612 (95.1%)	591 (94.6%)	525 (93.9%)	496 (97.1%)	0.04
Insulin	824 (48.6%)	277 (44.3%)	278 (49.7%)	269 (52.6%)	0.02
Diabetes self-care in the last 3 months, n (%)					
SMBG at least once weekly	920 (59.4%)	328 (57.5%)	311 (61.5%)	281 (59.4%)	0.43
Physical exercise at least 3 times per week	675 (41.3%)	253 (41.9%)	226 (41.9%)	196 (39.8%)	0.72
Adherence to balanced diet	1359 (81.1%)	501 (81.2%)	450 (81.8%)	408 (80.3%)	0.82
At least 2 self-care activities	1076 (63.5%)	396 (63.4%)	356 (63.7%)	324 (63.4%)	0.99
Metabolic targets, n (%)					
HbA_{1c} level <7.0% (53 mmol/mol)	552 (33.1%)	220 (35.8%)	175 (31.9%)	157 (31%)	0.19
BP <130/80 mmHg	476 (28.3%)	186 (30%)	139 (25%)	151 (29.8%)	0.11
LDL-cholesterol level <1.8 mmol/L	435 (27.7%)	157 (27.1%)	120 (23.2%)	158 (33.2%)	0.002
Triglyceride level <1.7 mmol/L	875 (53.2%)	319 (52.9%)	287 (52.6%)	269 (54.2%)	0.85
At least 3 treatment targets	576 (34.0%)	219 (35%)	175 (31.3%)	182 (35.6%)	0.26

Footnotes: Results are presented as mean (standard deviation) or number (percentage). General obesity was defined as BMI ≥25 kg/m^2. Hypertension was defined as blood pressure ≥130/80 mmHg and/or usage of blood pressure-lowering drugs. Dyslipidemia was defined as LDL-

© 2022 Chan JCN et al. *JAMA Network Open.*

cholesterol ≥1.8 mmol/L and/or usage of lipid-lowering drugs. ACR, albumin-to-creatinine ratio; BMI, body mass index (calculated as weight in kilograms divided by height in meter squared); BP, blood pressure; eGFR, estimated glomerular filtration rate; HbA_{1c}, glycated hemoglobin; HDL, high-density lipoprotein; LDL, low-density lipoprotein; RAAS, renin-angiotensin-aldosterone system; SMBG, self-monitoring of blood glucose.

© 2022 Chan JCN et al. *JAMA Network Open.*

eTable 10. Poisson regression to show the association of assignment to team-based empowered care compared with empowered care and usual care on attainment to multiple treatment targets at 12 months in the per-protocol population

	Model 1		Model 2	
	**RR (95% CI)**	**p-value**	**RR (95% CI)**	**p-value**
Team-based empowered care vs versus usual care (Reference)	1.24 (1.03-1.49)	0.02	1.27 (1.05-1.52)	0.01
Empowered care versus usual care (Reference)	0.95 (0.78-1.15)	0.58	0.95 (0.79-1.15)	0.62
Team-based empowered care versus empowered care (Reference)	1.31 (1.09-1.58)	0.004	1.33 (1.11-1.60)	0.002

Footnotes: The number of patients attaining at least 3 treatment targets was 231 (37.0%) in the usual care group, 199 (35.6%) in the empowered care group, and 213 (41.7%) in the team-based empowered care group. Results are presented as risk ratio (RR) with 95% confidence interval.
Model 1: adjusted for site
Model 2: Model 1 plus insulin use at baseline

© 2022 Chan JCN et al. *JAMA Network Open.*

eTable 11. Number of patients with incident clinical events by group randomization at 12 months in the per-protocol population

	Total	Usual care	Empowered care	Team-based empowered care	p-value
**Any events**					
Total	1120	420	369	331	
n (%)	75 (6.7%)	22 (5.2%)	26 (7.1%)	27 (8.2%)	0.27
**Cardiovascular disease**					
Total	1191	442	393	356	
n (%)	48 (4.0%)	14 (3.2%)	19 (4.8%)	15 (4.2%)	0.46
**Congestive heart failure**					
Total	1633	609	535	493	
n (%)	9 (0.6%)	4 (0.7%)	1 (0.2%)	4 (0.8%)	0.37
**End-stage kidney disease**					
Total	1695	625	559	511	
n (%)	36 (2.1%)	8 (1.3%)	13 (2.3%)	15 (2.9%)	0.14
**All-site cancer**					
Total	1612	601	530	481	
n (%)	9 (0.6%)	4 (0.7%)	3 (0.6%)	2 (0.4%)	0.92

Footnotes: n (%), number of patients (percentage). Any events are defined as the occurrence of cardiovascular disease, congestive heart failure, end-stage kidney disease and/or all-site cancer. Cardiovascular disease is defined as the occurrence of coronary artery disease, stroke and/or peripheral artery disease. End-stage kidney disease is defined as estimated glomerular filtration rate <15 mL/min/1.73m^2 or a need for kidney replacement therapy.

© 2022 Chan JCN et al. *JAMA Network Open.*

eTable 12. Number of patients with incident non-fatal clinical events by target attainment at 12 months in the per-protocol population

	Total	Attained <3 treatment targets	Attained ≥3 treatment targets	p-value
**Any events**				
Total	1120	701	419	
n (%)	75 (6.7%)	49 (7.0%)	26 (6.2%)	0.70
**Cardiovascular disease**				
Total	1191	746	445	
n (%)	48 (4.0%)	32 (4.3%)	16 (3.6%)	0.66
**Congestive heart failure**				
Total	1633	1107	616	
n (%)	9 (0.6%)	5 (0.5%)	4 (0.6%)	0.74
**End-stage kidney disease**				
Total	1695	1052	643	
n (%)	36 (2.1%)	25 (2.4%)	11 (1.7%)	0.45
**All-site cancer**				
Total	1612	1006	606	
n (%)	9 (0.6%)	6 (0.6%)	3 (0.5%)	1.00

Footnotes: n (%), number of patients (percentage). Any events are defined as the occurrence of cardiovascular disease, congestive heart failure, end-stage kidney disease and/or all-site cancer. Cardiovascular disease is defined as the occurrence of coronary artery disease, stroke and/or peripheral artery disease. End-stage kidney disease is defined as estimated glomerular filtration rate <15 $mL/min/1.73m^2$ or a need for kidney replacement therapy.

© 2022 Chan JCN et al. *JAMA Network Open.*

eFigure. Changes in the proportion of patients attaining at least 3 treatment targets at 12 months in the per-protocol population

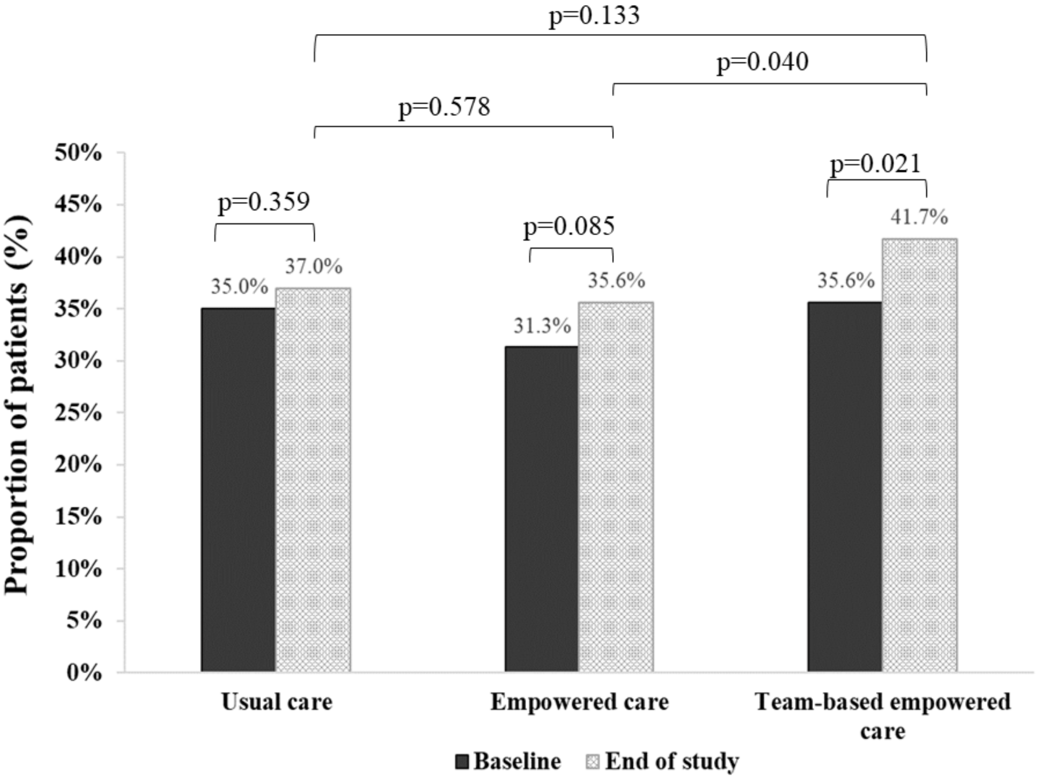

Footnotes: The McNemar test was used for within-group and χ^2 test was used for between-group comparisons of categorical variables.

© 2022 Chan JCN et al. *JAMA Network Open.*
