# Supplementary material for: Effect of a Web-Based Management Guide on Risk Factors in Patients With Type 2 Diabetes and Diabetic Kidney Disease: A JADE Randomized Clinical Trial
Source: JAMA Netw Open. 2022 Mar 25;5(3):e223862. doi: 10.1001/jamanetworkopen.2022.3862 (PMC8956973; doi:10.1001/jamanetworkopen.2022.3862)
Supplement: Supplement 3. — Data Sharing Statement [file jamanetwopen-e223862-s003.pdf]

Data Sharing Statement

Chan. Effect of a Web-Based Management Guide on Risk Factors in Patients With Type 2 Diabetes and Diabetic Kidney Disease. *JAMA Netw Open*. Published March 25, 2022. doi:10.1001/jamanetworkopen.2022.3862

Data
Data available: No

Additional Information
Explanation for why data not available: Data cannot be shared publicly as we did not have patients' consent to release the data in the public domain for open, unrestricted access. Researchers who are interested and meet the criteria for access to our data for research purpose may apply to Asia Diabetes Foundation (enquiry@adf.org.hk).
